# Computational Design of Broad-Spectrum Ebola Antibodies through Framework and Complementarity-Determining Region Synergistic Optimization

**DOI:** 10.34133/research.1211

**Published:** 2026-03-23

**Authors:** Xinhui Zhang, Xiuying Liu, Jingya Zhou, Peixiang Gao, Shengnan Pan, Xuehua Yang, Huarui Duan, Yi Liao, Fangyuan Zhang, Xuemeng Dong, Junyu Liu, Xiaojing Chi, Wei Yang

**Affiliations:** ^1^ Key Laboratory of Pathogen Infection Prevention and Control (Ministry of Education), National Institute of Pathogen Biology, Chinese Academy of Medical Sciences & Peking Union Medical College, Beijing, China.; ^2^ NHC Key Laboratory of Systems Biology of Pathogens, National Institute of Pathogen Biology, Chinese Academy of Medical Sciences & Peking Union Medical College, Beijing, China.; ^3^ State Key Laboratory of Respiratory Health and Multimorbidity, Chinese Academy of Medical Sciences & Peking Union Medical College, Beijing, China.

## Abstract

**Background:** A major challenge in neutralizing antibody development lies in balancing improved potency with broad-spectrum efficacy, particularly for highly mutable pathogens such as the Ebola virus (EBOV). We developed a computational–experimental pipeline that combines multi-parameter in silico predictions with targeted wet-lab validation to enhance the breadth and potency of 2 previously reported pan-EBOV antibodies, ADI-15878 and ADI-15946. **Methods:** This pipeline employs multiple computational tools to prioritize affinity-enhancing mutations in complementarity-determining regions (CDRs) and to explore indirect effects through framework region (FR) modifications. These include mCSM-AB and mmCSM-AB for mutant screening, Foldseek for FR grafting, and ZDOCK for complex modeling. Candidate antibodies were expressed, purified, and evaluated using pseudovirus neutralization assays, flow cytometry binding assays, and surface plasmon resonance (SPR) affinity measurements. **Result:** For ADI-15878, ‌a combination of FR grafting and CDR mutagenesis generated the variant W32G-LC, which maintained structural integrity while improving neutralization against EBOV, Bundibugyo virus (BDBV), and Sudan virus (SUDV)—showing approximately 17-fold, 7-fold, and 2-fold enhancements over the parental antibody, respectively. For ADI-15946, ‌docking-guided deep mutation scanning‌ enabled the design of a multi-site light chain variant (H27Q/S52Y/G66R-LC and A50Y/S52Y/L54R-LC‌), which conferred greater than 40-fold and 100-fold increases in neutralization against SUDV, respectively, without compromising activity against EBOV/BDBV. Improved neutralization correlated with increased buried surface area (BSA) and enhanced SPR-measured affinities. **Conclusions:** The integrated FR–CDR optimization strategy combined with docking-guided multi-site design facilitates the rapid generation of antibody variants with improved breadth and potency. This modular pipeline provides a practical approach for updating therapeutic antibodies to combat diverse or emerging viral variants.

## Introduction

**Fig. 1.**
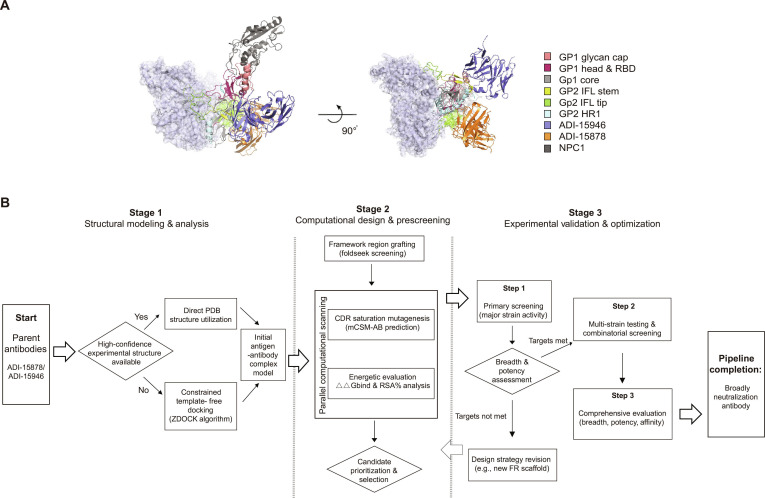
Ebola virus (EBOV) antibody evolution through structure-based computation. (A) Structural representation of the EBOV GP trimer in complex with antibodies ADI-15878, ADI-15946, and receptor NPC1. ADI-15878 engages the GP1 N-terminal pocket and the GP2 fusion loop; ADI-15946 targets the GP1 3_10_ pocket, the GP2 N terminus, and the GP2 fusion loop; NPC1 binds the GP1 RBD region. (B) Schematic of the integrated computational–experimental pipeline for broad-spectrum antibody optimization. The workflow illustrates the iterative, closed-loop strategy employed to enhance the potency and breadth of anti-EBOV antibodies ADI-15878 and ADI-15946. The pipeline comprises 3 core, interdependent stages.

Ebola virus (EBOV), a member of the *Filoviridae* family, is the primary causative agent of Ebola virus disease (EVD) [1]. Recurring regional EBOV outbreaks between 2013 and 2022 have solidified EBOV as a global public health threat. Six EBOV species have been identified: Zaire (EBOV), Sudan (SUDV), Tai Forest (TAFV), Bundibugyo (BDBV), Reston (RESTV), and Bombali (BOMV), among which EBOV, BDBV, and SUDV exhibit particularly high pathogenicity [2,3]. Viral entry is mediated by the EBOV glycoprotein (GP), which interacts with host cell receptors, such as NPC1 and TIM-1, through receptor-binding domains (RBDs) [4,5]. The GP proteins comprise 2 subunits: GP1, containing glycan cap, RBD, and core structural regions, and GP2, which includes fusion peptide and heptad repeat regions (Fig. 1A). These subunits form disulfide-linked heterodimers that assemble into trimeric structures [6].

Several antibody-based therapeutics have been developed. The U.S. Food and Drug Administration (FDA)-approved cocktail Inmazeb (REGN-EB3) consists of antibodies targeting the RBD, glycan cap, and internal fusion loop (IFL) of GP, respectively [7]. Ansuvimab (mAb114) specifically blocks the GP RBD, inhibiting viral interaction with the host cell receptor NPC1 [8]. The ZMapp and MIL77 cocktails incorporate antibodies targeting different GP regions: c13C6 (MIL77-3) recognizes the glycan cap, whereas c2G4 (MIL77-1) and c4G7 (MIL77-2) target the GP base [9,10]. Although these therapies show efficacy against EBOV [11], their activity against other EBOV species remains limited.

Antibody therapy has become a cornerstone strategy for combating outbreaks of highly pathogenic viruses [12,13]. Potent neutralizing antibodies can block viral entry, reduce mortality, and promote recovery. Such antibodies are typically sourced from convalescent patients or engineered through animal immunization and synthetic library screening [14,15]. However, the unpredictable nature of outbreaks calls for efficient and ethical development methods. A major challenge is viral evolution under immune pressure, which can lead to neutralization escape or reduced efficacy against certain strains. This underscores the need for continuous advancement of antibody optimization technologies to improve broad-spectrum coverage and counteract immune evasion.

While traditional antibody development remains time-intensive, computational approaches now enable in silico optimization through diverse modeling strategies [16,17]. These methods allow targeted enhancement of neutralizing epitopes and cross-species affinity, thereby improving both neutralizing potency and breadth [18,19]. In recent years, the integration of computational biology and artificial intelligence (AI) has shifted antibody engineering from empirical screening toward rational design [20,21]. Advances progress along multiple fronts: Generative AI, particularly diffusion models, has opened new possibilities for de novo antibody sequence and structure generation [22]. Concurrently, the rational optimization of existing antibodies has matured into a mainstream application, driven primarily by computational prediction of affinity-enhancing mutations, often achieved through precise complementarity-determining region (CDR) modifications and exploration of framework region (FR) effects. Computational design strategies have also been extended to specialized scaffolds such as nanobodies [23], with emphasis on establishing a robust “design-validate” closed-loop systems to improve translational outcomes. Nevertheless, current computational methods still face challenges in prediction accuracy, conformational dynamics assessment, and consistency across different models. This highlights the importance of incorporating multi-method cross-validation and uncertainty quantification into the design pipeline.

Neutralizing antibodies such as ADI-15878 and ADI-15946 have demonstrated promising cross-species reactivity in preclinical studies [24], yet further optimization of their potency and breadth is needed to achieve truly pan-EBOV coverage. Computational simulation offers a promising avenue to accelerate this process. Here, we propose an integrated optimization framework that combines computational modeling with wet-lab validation. The workflow leverages full antibody–antigen complex structures to guide iterative development: Computational predictions inform experimental testing, and experimental results in turn refine the predictive models [25]. This study aims to enhance neutralizing activity and broad-spectrum efficacy through a streamlined and rapid methodology.

## Results

### Development of an optimization pipeline for EBOV neutralizing antibodies‌

Using the pan-EBOV neutralizing antibodies ADI-15878 and ADI-15946 as model candidates, we employed computational strategies to optimize their binding affinity and antiviral activity. ADI-15878 mediates broad-spectrum neutralization by targeting both the GP1 N-terminal pocket and the GP2 fusion loop (Fig. 1A). Although it exhibits broad activity, ADI-15878 monotherapy conferred only partial protection in animal models, suggesting room for improvement. ADI-15946 binds the GP1 3_10_ pocket, GP2 N terminus, and the GP2 fusion loop (Fig. 1A) and shows strong neutralization against EBOV and BDBV but limited activity against SUDV. Given that increased antibody binding affinity to EBOV GP can enhance neutralization potency and potentially lower clinical dosing, we aimed to computationally improve either the neutralizing potency or breadth of these antibodies.

To this end, we established a computational pipeline that leverages advances in computational algorithms simulating antigen–antibody interaction to emulate in silico directed antibody evolution (Fig. 1B). This framework replaces conventional variable-region mutant phage display with a deep mutation scanning (DMS) system. All candidate variants were prioritized based on a predefined multi-parameter scoring scheme that integrated predicted binding free energy change (ΔΔG_bind_), relative solvent accessibility (RSA%), and interface localization. These criteria were established a priori and applied consistently across all design stages. By integrating computational predictions with targeted experimental validation, we aimed to identify variants with improved broad-spectrum neutralization. The pipeline begins with systematic analysis of the antigen–antibody interface (stage 1). Experimentally determined complex structures from the Protein Data Bank (PDB) are used when available; otherwise, homologous or computationally predicted structures are employed. In cases where no complex structure exists, antibody models are docked against GP to simulate binding. Based on these models, computational saturation mutagenesis is performed by introducing single-point mutations across the binding region (stage 2). Mutations predicted to enhance function are carried forward into multiple rounds of pseudovirus neutralization screening. Finally, improvements in binding affinity are confirmed using fluorescence-activated cell sorting (FACS) or surface plasmon resonance (SPR) (stage 3).

### ADI-15878 CDR optimization and FR grafting‌

**Fig. 2.**
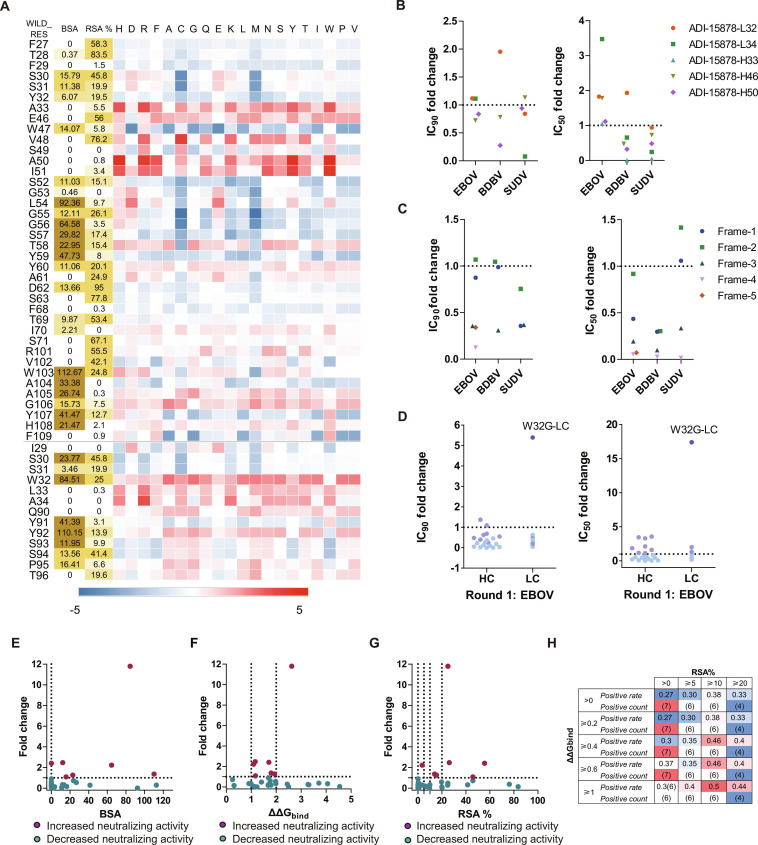
Computational modification of ADI-15878 for pseudovirus neutralization‌. (A) Calculate single-point mutation scans. RSA% denotes relative accessible surface area. Red indicates positive values; blue denotes negative values. Deeper red indicates higher ΔΔG_bind_ values, reflecting greater predicted affinity gains. (B) Effect of CDR mutations on EBOV, BDBV, and SUDV pseudovirus neutralization. (C) Effect of FR mutations on pseudovirus neutralization. (D) Effect of combining CDR mutations with Frame-2. Light colors indicate new antibodies with IC_50_ values higher than ADI-15878, while dark colors indicate IC_50_ values lower than ADI-15878. Data in (B) to (D) represent fold changes in IC₅₀/IC₉₀ relative to parental ADI-15878. (E) Correlation between BSA values at mutant amino acid sites and antigen-specific neutralization activity. (F) Correlation between predicted average ΔΔG_bind_ and neutralization fold change. (G) Effect of average RSA% on neutralization fold change. (H) ΔΔG_bind_ and RSA% scan range design. The table contains Positive rate (Positive count).

We analyzed the binding interface between ADI-15878 and EBOV-GP using PDB entries 6DZL and 6EA5, with emphasis on the buried surface area (BSA) and interacting residues. Key antigen contacts were mediated by light chain CDR1 and CDR3, and heavy chain CDR1, CDR2, and CDR3. Heavy chain residues L54 and Y59 were critical for hydrogen bonding with GP2, while residues Y32, L54, G55, G56, S57, T58, T69, I70, and W103 exhibited high BSA values with GP1. A computational single-point mutation scan was performed on BSA > 0 and neighboring residues (Fig. 2A), thereby restricting candidate mutations to interface-localized positions. Based on predicted affinity gains, we selected the following mutations: W32G and A34R in the light chain, and A33R, E46M, and A50H in the heavy chain for experimental verification. This initial selection was intended to evaluate the predictive value of single-point energetic screening rather than to generate optimized multi-point variants. Pseudovirus neutralization assays against EBOV, BDBV, and SUDV revealed that light chain A34R and heavy chain A33R substantially impaired neutralization. Other mutations conferred modest improvements against EBOV relative to wild-type ADI-15878. Only the ADI-15878-L32 variant showed slightly enhanced activity against BDBV. Against SUDV, all mutants underperformed compared to the wild-type antibody (Table 1 and Fig. 2B). Although the original residues showed BSA = 0, introducing bulky or charged side chains increased local BSA and disrupted the native binding interface, thereby compromising functional activity, highlighting the limitations of affinity-driven single-parameter optimization.

**Table 1.** ADI-15878 CDR mutations affect neutralizing activity against 3 Ebola viruses

	EBOV				BDBV				SUDV			
	IC_90_	Fold change	IC_50_	Fold change ^c^	IC_90_	Fold change	IC_50_	Fold change	IC_90_	Fold change	IC_50_	Fold change
	(μg/ml) ^a^		(μg/ml) ^b^		(μg/ml)		(μg/ml)		(μg/ml)		(μg/ml)	
ADI-15878	1.52	1	0.48	1	0.07	1	0.01	1	0.9	1	0.04	1
ADI-15878-L32	1.36	1.12	0.26	1.83	0.04	1.95	0.01	1.94	1.07	0.84	0.05	0.94
ADI-15878-L34	1.37	1.11	0.14	3.47	/	/	0.02	0.66	11.36	0.08	0.18	0.24
ADI-15878-H33	/	/	0.45	1.05	/	/	5.26	0	/	/	0.96	0.05
ADI-15878-H46	2.1	0.72	0.27	1.78	0.09	0.78	0.02	0.48	0.79	1.13	0.06	0.73
ADI-15878-H50	1.82	0.84	0.43	1.12	0.25	0.28	0.04	0.32	0.96	0.94	0.09	0.48

^a^ IC_50_ denotes the antibody concentration required to inhibit 50% of the luciferase signal expression following pseudovirus infection (μg/ml).

^b^ IC_90_ denotes the antibody concentration required to inhibit 90% of the luciferase signal expression following pseudovirus infection (μg/ml).

^c^ Fold change denotes the multiple by which ADI-15878’s IC_50_ exceeds that of the mutant antibody, or by which ADI-15878’s IC_90_ exceeds that of the mutant antibody.

The binding interface between ADI-15878 and GP involves key residues on the heavy chain (S30, S31, S52, G53, L54, G56, S57, Y59, W98, A99, and Y102) and on the light chain (S30, W32, Y91, Y92, S93, and S94). When the light chain undergoes the A34R mutation, the newly introduced arginine side chain enhances hydrophobic interactions with Y91, thereby disrupting the π–π network formed between Y91 and L92 of the light chain and H103 of the heavy chain. These residues are critical for maintaining the GP-binding interface. Similarly, the A33R substitution on the heavy chain strengthens local interactions with A99 and Y102, both of which are key residues involved in antigen recognition. These findings suggest that excessive or improperly positioned charged residues can perturb the local geometry of densely packed interfacial regions, consistent with the structural constraints inferred from the computational models. Therefore, simply expanding the contact area does not necessarily enhance binding affinity, and effective optimization requires balancing contact density with spatial complementarity, as reflected by the combined ΔΔG_bind_ and structural filtering strategy used in subsequent designs.

Collectively, our initial attempts at interface optimization alone did not achieve balanced improvements. To overcome this limitation, we turned to FR grafting as a strategy to modulate CDR geometry while preserving the canonical antigen-binding site. Using Foldseek, we grafted and evaluated 5 novel FRs. Neutralization assays with Ebola pseudoviruses revealed that these FR modifications altered antibody activity (Fig. 2C). Among the candidates, Frame-2 was selected for further optimization because it induced minimal changes in neutralization against pathogenic Ebola strains. This scaffold was therefore chosen as a neutral structural background for subsequent CDR-focused optimization. Structural predictions generated with AlphaFold3 (AF3) showed a root mean square deviation (RMSD) of ~0.7 Å relative to the parental antibody (Fig. S1) and excellent human nativeness (Fig. S2), confirming overall structural preservation and suggesting FR compatibility with the prototype during GP binding.

To further enhance activity, we performed a single-point mutation scan targeting GP-binding residues of ADI-15878 (Fig. 2A). These mutations were introduced into Frame-2, generating 34 variants. Primary screening against EBOV pseudovirus identified 9 antibodies with improved IC_50_ (half-maximal inhibitory concentration) values, 3 of which also showed enhanced IC_90_ (90% inhibitory concentration) (Table 2 and Fig. 2D). Computational and experimental analysis revealed that mutations at antibody–antigen contact sites exerted the most pronounced effect on neutralization. Simulations indicated that residues with a BSA > 0 were highly likely to enhance neutralizing activity (Fig. 2E). Consistently, by restricting candidate mutations to such interface-exposed residues with favorable RSA% and ΔΔG_bind_ values, we substantially enriched for variants that demonstrated experimentally validated improvements in neutralization potency (Fig. 2F to H).

**Table 2.** CDR mutations applied to Frame-2 affect neutralizing activity against EBOV

Name	HC/LC ^a^	IC_50_ (μg/ml) ^b^	Fold change ^c^	IC_90_ (μg/ml) ^d^	Fold change	RSA % ^e^	PRED_△△G ^f^	Name	HC/LC	IC_50_ (μg/ml)	Fold change	IC_90_ (μg/ml)	Fold change	RSA %	PRED_△△G
T28W	HC	1.40	0.21	5.49	0.10	83.5	0.31	I29D	LC	0.22	1.33	2.86	0.19	0	1.691
S30E	HC	0.18	1.60	1.15	0.48	45.8	1.17	S30N	LC	0.32	0.89	1.42	0.39	45.8	0.251
A33R	HC	/	/	/	/	5.5	3.309	S31N	LC	1.92	0.15	5.94	0.09	19.9	0.313
E46M	HC	0.95	0.31	4.50	0.12	56	2.012	W32G	LC	0.02	17.39	0.10	5.39	25	2.622
V48C	HC	0.54	0.54	1.21	0.45	76.2	3.712	L33R	LC	0.74	0.39	2.08	0.27	0.3	2.444
S49R	HC	0.61	0.47	2.42	0.23	0	1.99	A34R	LC	0.83	0.35	3.39	0.16	0	3.272
A50H	HC	4.41	0.07	21.00	0.03	0.8	4.542	Q90M	LC	1.16	0.25	10.03	0.05	0	1.791
I51W	HC	0.63	0.46	10.17	0.05	3.4	4.06	Y91W	LC	0.67	0.43	6.10	0.09	3.1	0.992
L54D	HC	16.33	0.02	222.77	0.00	9.7	2.498	Y92G	LC	0.14	2.00	1.17	0.47	13.9	1.798
G55D	HC	0.09	3.30	0.80	0.68	26.1	1.161	S93G	LC	0.60	0.48	1.16	0.47	9.9	0.989
G56H	HC	0.14	2.11	1.40	0.39	3.5	1.095	S94G	LC	0.61	0.47	1.75	0.31	41.4	0.908
T58G	HC	0.16	1.85	0.50	1.09	15.4	1.932	P95M	LC	1.19	0.24	10.91	0.05	6.6	1.678
Y60W	HC	4.32	0.07	19.32	0.03	20.1	1.122	T96M	LC	0.33	0.88	0.88	0.63	19.6	1.761
A61E	HC	0.50	0.58	1.51	0.36	24.9	1.409	H58-L92		0.03	11.29	0.32	1.74		
I70W	HC	20.18	0.01	190.15	0.00	0	1.231	H101-L92		0.05	5.49	0.51	1.09		
R101N	HC	0.08	3.56	0.40	1.37	55.5	1.708	H107/108-L92		1.68	0.17	/	/		
W103H	HC	0.53	0.54	3.06	0.18	24.8	1.76	H30-L32		0.04	7.13	0.37	1.51		
A105G	HC	0.36	0.81	2.12	0.26	0.3	1.977	H58-L32		8.26	0.03	344.78	0.00		
AG105/106GA	HC	0.25	1.16	2.26	0.24	0.3/7.5	1.977/2.283	H101-L32		0.02	11.70	0.33	1.68		
YH107/108HY	HC	0.08	3.45	0.88	0.62	12.7/2.1	1.116/1.252	H30/58-L32		0.03	8.49	0.49	1.13		
F190W	HC	0.26	1.13	1.01	0.54	0.9	1.681	H30/101 L32		0.10	2.88	1.35	0.41		
H30/58-L		0.35	0.83	0.45	1.22			H58/101-L32		0.01	23.66	0.20	2.77		
H58/101-L		0.16	1.84	0.42	1.31			H30/58/101-L32		1.21	0.24	11.08	0.05		
H30/58/101-L		0.20	1.43	0.54	1.01			ADI15878		0.29	1.00	0.55	1.00		

^a^ HC/LC denotes heavy chain CDR region/light chain CDR region.

^b^ IC_50_ denotes the antibody concentration required to inhibit 50% of the luciferase signal expression following pseudovirus infection (μg/ml).

^c^ Fold change indicates the multiple by which ADI-15878’s IC_50_ exceeds that of the mutant antibody, or by which ADI-15878’s IC_90_ exceeds that of the mutant antibody.

^d^ IC_90_ denotes the antibody concentration required to inhibit 90% of the luciferase signal expression following pseudovirus infection (μg/ml).

^e^ RSA% denotes the relative accessible surface area.

^f^ PRED_△△G means ΔΔG_bind_, predicted change in binding affinity; higher values indicate more marked affinity enhancement.

### Enhanced activity and affinity of ADI-15878 via the W32G light chain mutation‌

**Fig. 3.**
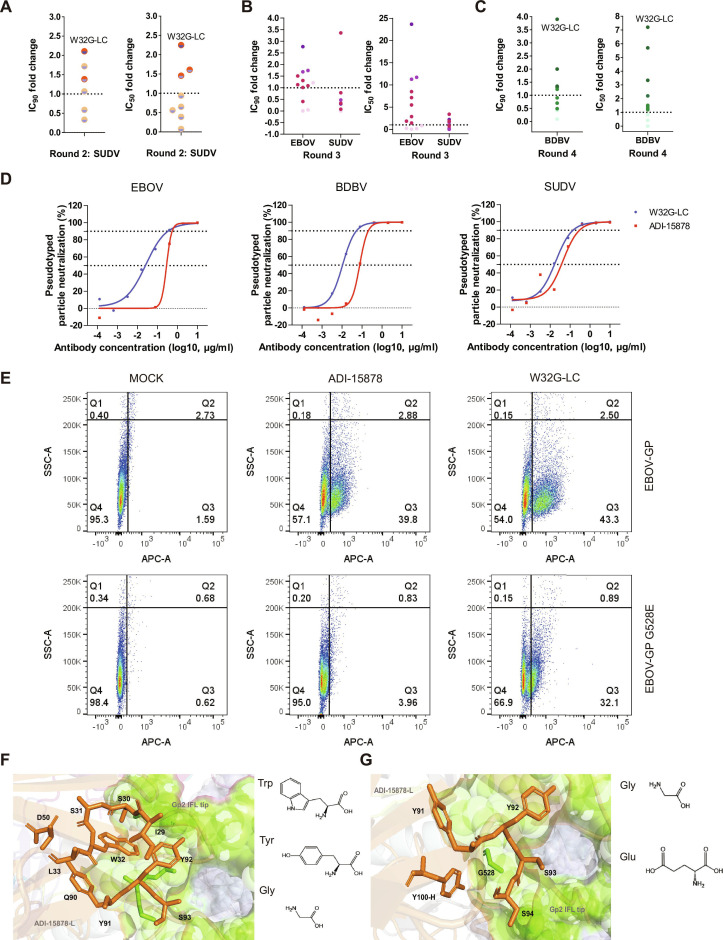
Comprehensive characterization of ADI-15878 variants. (A) Single-point CDR mutations on the Frame-2 scaffold assessed for SUDV neutralization (IC_50_, IC_90_). The circular dots indicate the fold reduction in IC_50_ and IC_90_ values for the mutant compared to ADI-15878. Half of the dots are blue, indicating enhanced neutralizing activity against EBOV. The other half are orange, with lighter shades representing new antibodies with IC_50_ values higher than ADI-15878, and darker shades indicating IC_50_ values lower than ADI-15878. (B) Neutralization profiles of multi-mutation variants against EBOV and SUDV. (C) BDBV neutralization profiles of selected variants. The circular dots in (B) and (C) indicate the fold reduction in IC_50_ and IC_90_ values for the mutant compared to ADI-15878. Light colors indicate new antibodies with IC_50_ values higher than ADI-15878, while dark colors indicate IC_50_ values lower than ADI-15878. (D) Neutralization curves of W32G-LC and ADI-15878 against EBOV, BDBV, and SUDV. (E) Flow cytometry binding analysis to wild-type and G528E mutant GP. Mean fluorescence intensity (MFI) values were normalized to the negative control. In the absence of GP recognition, cells accumulated in the Q4 region; antibody binding shifted cells toward Q3. (F) Structural environment of light chain residue W32 (PDB:6DZL), and Trp 32_WT_, Tyr 92_WT_, and Gly32/92_MUT_ are shown on the right. (G) Location of GP escape site G528 relative to antibody paratope residues, and Gly528_WT_ and Glu528_MUT_ are shown on the right.

Following FR grafting, a second round of broad-spectrum antibody optimization was conducted using SUDV pseudovirus. The results showed that the G55D-HC mutation led to a complete loss of neutralizing activity against SUDV, while YH107/108HY-HC and Y92G-LC exhibited markedly reduced neutralization. In contrast, G56H-HC and F190W-HC caused only minor reductions in SUDV neutralization. Ultimately, we identified 4 single-point mutant antibodies that retained high activity against both EBOV and SUDV (Figs. 2D and 3A). These findings indicate that synergistic optimization of FR and CDR regions can enhance antibody potency without compromising cross-species activity.

To explore whether beneficial mutations could act synergistically, we introduced combinations of these mutations in a third round of screening. Neutralization experiments with EBOV and SUDV showed that some combined mutants exhibited improved neutralization activity against EBOV (Fig. 3B and Table 2). However, enhancement against SUDV was marginal, and in some cases, neutralization activity against SUDV was substantially reduced (Fig. 3B and Table 3). Notably, for broad-spectrum neutralizing antibodies, excessive increases in binding affinity toward a specific antigen may disrupt the balance of epitope recognition across different virus strains. Therefore, during computational analysis, a comprehensive evaluation of affinity changes across multiple antigen–antibody interactions is essential to preserve broad-spectrum properties.

**Table 3.** CDR mutations applied to Frame-2 affect neutralizing activity against SUDV

Name	HC/LC ^a^	IC_50_ (μg/ml) ^b^	Fold change ^c^	IC_90_ (μg/ml) ^d^	Fold change	Name	IC_50_ (μg/ml)	Fold change	IC_90_ (μg/ml)	Fold change
S30E	HC	0.04	0.94	0.17	1.72	H58-L92	1.27	0.03	/	/
G55D	HC	/	/	/	/	H101-L92	2.62	0.01	/	/
G56H	HC	0.07	0.56	0.89	0.34	H58/101-L	0.01	3.40	0.09	3.36
T58G	HC	0.03	1.46	0.28	1.07	H30/58/101-L	0.05	0.80	/	/
R101N	HC	0.02	1.61	0.21	1.38	H30-L32	0.02	1.79	0.38	0.78
YH107/108HY	HC	0.10	0.39	/	/	H101-L32	0.02	1.53	0.63	0.47
F190W	HC	0.06	0.65	0.51	0.59	H30/58-L32	0.02	1.83	0.90	0.33
W32G	LC	0.02	2.25	0.14	2.11	H30/101 L32	0.14	0.26	3.80	0.08
Y92G	LC	0.49	0.08	/	/	H58/101-L32	0.02	2.21	0.99	0.30
ADI-15878		0.04	1.00	0.30	1.00	H30/58/101-L32	0.64	0.06	209.96	0.00

^a^ HC/LC denotes heavy chain CDR region/light chain CDR region.

^b^ IC_50_ denotes the antibody concentration required to inhibit 50% of the luciferase signal expression following pseudovirus infection (μg/ml).

^c^ Fold change denotes the multiple by which ADI-15878’s IC_50_ exceeds that of the mutant antibody, or by which ADI-15878’s IC_90_ exceeds that of the mutant antibody.

^d^ IC_90_ denotes the antibody concentration required to inhibit 90% of the luciferase signal expression following pseudovirus infection (μg/ml).

In the fourth round of screening, BDBV pseudoviruses were used to select the final single-point and combination mutants (Fig. 3C). We successfully identified the W32G-LC antibody, which broadly neutralizes 3 highly pathogenic EBOVs. This antibody exhibited a significant increase in neutralization activity against a computationally simulated EBOV template, a 7-fold improvement against BDBV, and retained the parental antibody’s neutralization activity against SUDV with a 2-fold enhancement (Fig. 3D).

We next evaluated changes in binding affinity. Since the ADI-15878 binding site lies between 2 GP protomers in the Ebola GP trimer (Fig. 1A) and purified trimeric GP was difficult to obtain, we used FACS to measure antibody binding to GP proteins expressed in 293T cells. In the absence of antibody, cells clustered in the Q4 region (Fig. 3E and Fig. S3); antibody addition shifted the population rightward into the Q3 region. At 0.08 μg/ml, a significant difference in cell counts in Q3 was observed between W32G-LC antibody and parental ADI-15878, indicating stronger GP binding by W32G-LC at lower concentrations (Fig. 3E and Fig. S3). In addition, previous studies have shown that the G528E mutation on GP is a critical site for EBOV escape from ADI-15878 neutralization [26]. At 10 μg/ml, ADI-15878 binding to EBOV-GP G528E was drastically reduced (only 4% of cells in Q3 region), whereas W32G-LC showed marked improvement, with over 30% of cells shifting to Q3 (Fig. 3E and Fig. S3). Nevertheless, G528E remains a key binding site for W32G-LC. These results indicate that the enhanced neutralizing activity of W32G-LC arises from improved GP engagement rather than alteration of the canonical binding site.

Analysis of the simulated structures provides mechanistic insight. In the parental antibody, light chain residue W32 interacts with S30, S31, L33, D50, Y91, and Y92 on light chain and L529, A530, and F535 on GP2 within 4 Å. Both W32 and Y92 on light chain interact with the GP2 fusion loop pocket (Fig. 3F). Upon mutation to G32, Y92 no longer interacts with residue 32. In Frame-2, we introduced S51Y and S53N mutations, which are predicted to influence interactions between D50 and neighboring residues, including S31, W32/G32, L33, S49, A51, S52, S53, and Y91. Thus, Frame-2 indirectly modulates interactions between G32, Y92, and the GP protein. Since W32G-LC and Y92G-LC enhance EBOV neutralization, we hypothesize that weakening the W32-Y92 interaction could further improve activity. However, Y92 is critical for maintaining the broad-spectrum efficacy (Tables 2 and 3). The W32G mutation enlarges the interaction space between Y92 and GP. Although the ADI-15878 escape site G528 does not contribute to interaction forces, it is located near a benzene ring cluster formed by antibody light chain residues Y91, Y92, S93, and S94, and heavy chain residue Y100 within 4 Å (Fig. 3G). Once the G528 mutates to E528, the extended side chain interacts with this area, affecting the interactions between neighboring amino acids. We propose that W32G reduces the interaction strength between Y92 and the benzene ring cluster, thereby mitigating the disruptive effect of the G528E substitution and partially restoring recognition.

We performed a comprehensive structural analysis using AF3 to obtain quantitative metrics that support the FR–CDR synergistic effects. The AF3 predictions show that the docking conformation of the EBOV-GP protein with the ADI-15878 antibody aligns well with the experimentally determined structure 6DZL from the PDB database (Fig. S4). The predicted model achieved an interface predicted template modeling score (ipTM) of 0.78 and a pTM of 0.81, with a combined score above 0.75, indicating high confidence in the overall structure and docking orientation of the ADI-15878–GP complex. Using the same AF3-based approach, we also predicted the docking of W32G-LC with EBOV GP (Fig. S4). This model displayed an ipTM of 0.76 and a pTM of 0.79, again with a combined score above 0.75, supporting its structural reliability. Structural alignment with 6DZL confirms that W32G-LC binds EBOV GP at a position consistent with ADI-15878, indicating that the introduced mutation preserves the canonical binding mode. Notably, structural comparison revealed a marked conformational rearrangement in the HCDR1 region of W32G-LC relative to ADI-15878. This change resulted in more than 24-fold increase in the BSA, from 1.12 Å^2^ to 27.07 Å^2^, and recruited previously non-interacting residues into the binding interface (Fig. S4). This expansion created space for new favorable interactions, including contacts with the glycosylation site N204. Importantly, N204 is a key glycosylation site on GP, and the enhanced interaction between the antibody and this glycosylated region is likely to strengthen binding and broaden neutralization. In comparison, other variants showed distinct interface profiles: Y92G-LC: The light chain CDR3 interface decreased from 19.24 Å^2^ to 14.00 Å^2^, while its CDR1 interface remained modest (7.28 Å^2^) and did not establish new advantageous epitopes. S31N-LC: Displayed only a limited CDR1 interface increase to 9.69 Å^2^ (Fig. S5).

### Computation-aided optimization enhances the broad-spectrum activity of ADI-15946

ADI-15946 neutralizes EBOV and BDBV but shows weak activity against SUDV. To improve SUDV neutralization, we employed computational design. While structural data are available only for the ADI-15946–EBOV-GP complex in PDB, AF3 predictions of this interface deviated substantially from the experimental structure (Fig. S6). All alignment results outperformed the AF3 prediction results. To ensure the objectivity of the predicted complex models (Figs. S7 to S10), we selected ZDOCK over HDOCK between the 2 docking strategies. HDOCK’s performance heavily relies on known homologous templates [27,28], whereas ZDOCK employs a template-independent global search algorithm that performs de novo predictions based solely on shape complementarity and physicochemical properties. This study aims to explore novel interaction patterns between antibodies and different EBOV GP variants. Given the scarcity of high-quality templates for certain targets, ZDOCK’s bias-free approach and its ability to discover novel binding conformations align more closely with our research objectives.

We therefore generated docking models of ADI-15946 bound to SUDV-GP as a reference for the next step, affinity optimization. Molecular docking with EBOV-GP reproduced results consistent with the experimental PDB structure (6MAM), validating our approach. To further evaluate the docking model, we performed molecular dynamics-based feasible transition pathway analysis, using the docked complex as the initial structure and 6MAM as the reference. The final fitted models showed RMSD values of 0.2 Å relative to the reference, indicating that the docked complex represents a static snapshot within the natural oscillatory range of the antigen–antibody interaction (Fig. S9). We then applied this approach to dock ADI-15946 with SUDV-GP (Fig. S10).

**Fig. 4.**
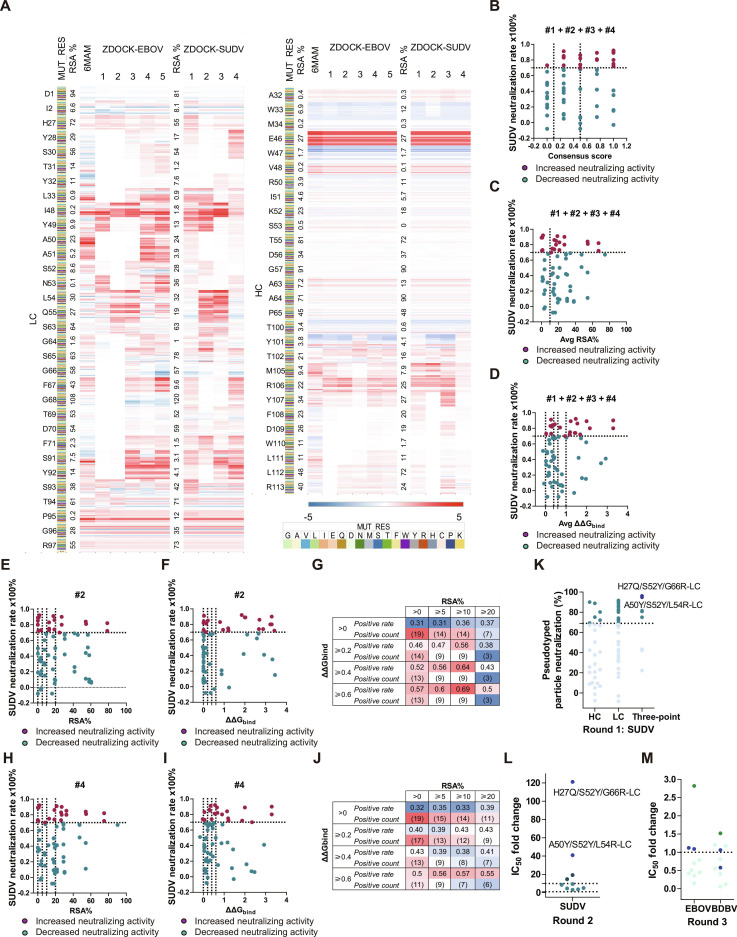
Computational enhancement of ADI-15946 breadth and pseudovirus neutralization‌. (A) Computational scanning of single-point mutations. RSA% indicates relative accessible surface area. Red indicates positive values; blue denotes negative values. (B) Correlation between multi-template consensus and neutralization activities. Each predicted ΔΔG_bind_ at this site exceeds 0.6, so we propose averaging the scores and adding 0.25. (C) Correlation between predicted average ΔΔG_bind_ and neutralization activities. (D) Effect of average RSA% on neutralization. (E) Effect of RSA% on neutralization in SUDV–GP–ADI-15946 model 2. (F) Relationship between predicted ΔΔG_bind_ and neutralization in SUDV–GP–ADI-15946 model 2. (G) ΔΔG_bind_ and RSA% scan range design in SUDV–GP–ADI-15946 model 2. (H) Effect of RSA% on neutralization in SUDV–GP–ADI-15946 model 4. (I) Relationship between predicted ΔΔG_bind_ and neutralization in SUDV–GP–ADI-15946 model 4. (J) ΔΔG_bind_ and RSA% scan range design in SUDV–GP–ADI-15946 model 4. (K) Neutralization of SUDV pseudovirus by ADI-15946 variants at 10 μg/ml. The circular dots indicate the percentage of neutralization by mutants against SUDV, while dark colors indicate a higher neutralization capacity than ADI-15946. The dark blue dot indicates the antibody with the strongest neutralizing capacity. (L) IC₅₀ fold changes of selected mutants against SUDV. The circular dots indicate the fold reduction in IC_50_ values for the mutant compared to ADI-15946, while dark colors indicate a higher neutralization capacity than ADI-15946. The dark blue dot indicates the antibody with the strongest neutralizing capacity. (M) Neutralization breadth of lead variants against EBOV and BDBV. Dark blue dots highlight the most potent multi-point mutants (H27Q/S52Y/G66R-LC, A50Y/S52Y/L54R-LC).

To validate the docking model, we performed single-point mutation scans on both experimental PDB and docked structures (Fig. 4A and Fig. S11), assessing consistency of changes in antibody–antigen interaction energies. The results generally supported the model’s validity, although minor discrepancies were observed, attributable to inherent docking biases and structural variations at the SUDV-GP antigenic site relative to the PDB templates. This confirmed that docking predictions provide useful, though not absolute, guidance (Fig. 4B to D and Fig. S12). Based on the single-point mutational landscape (Fig. 4E to J and Fig. S13), multi-point variants were constructed using mmCSM-AB (Fig. S14). Importantly, combinatorial designs were restricted to filtered single-point mutations with favorable energetic and structural properties, rather than exhaustive enumeration of all possible combinations. We therefore expanded our selection criteria: first, choosing mutation sites that showed consistent trends across 10 different models; second, lowering the RSA% threshold from ≥10 to >0; and third, combining single-point mutation prediction results with neutralization assay results for threshold screening and high-confidence template selection. We incorporated multi-point mutation simulations to avoid antagonistic effects from combining single-point variants. Importantly, multi-point variants were not generated by exhaustive combinatorial enumeration. Instead, designs were constrained to a filtered subset of the aforementioned single-point mutations that met predefined energetic (ΔΔG_bind_) and structural (RSA%/BSA and model consistency) criteria. We evaluated affinity changes per model group and identified 64 high-confidence single-point mutations. Based on these results, a limited number of multi-point variants (*n* = 8) were constructed from the filtered single-point set using mmCSM-AB to capture potential synergistic effects, rather than by exhaustive combinatorial sampling (Fig. 4K and Table S1).

**Fig. 5.**
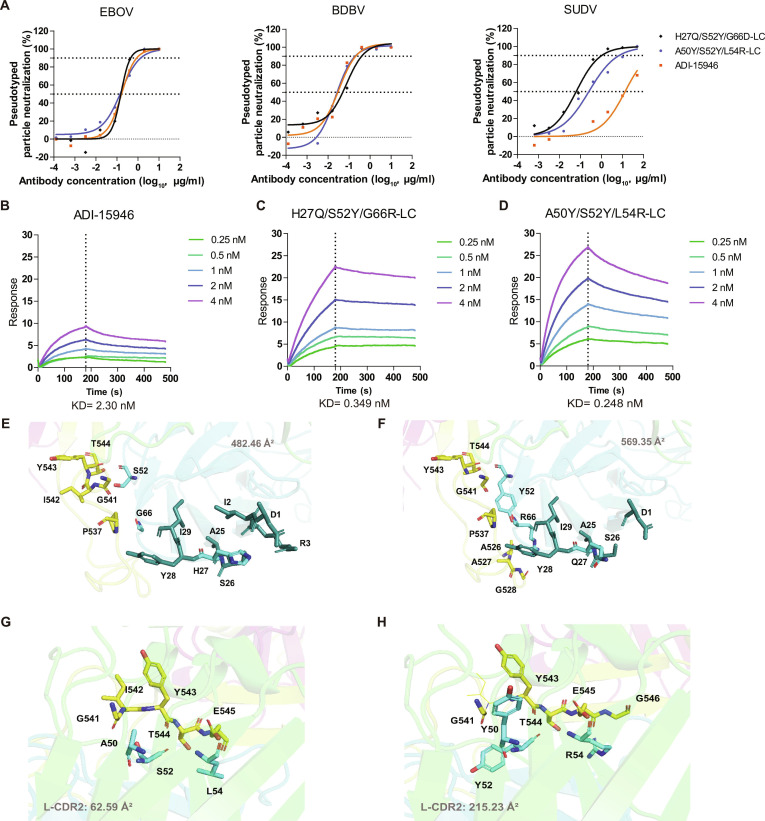
Affinity increase in H27Q/S52Y/G66R-LC and A50Y/S52Y/L54R-LC mutants. (A) Neutralization curves against EBOV, BDBV, and SUDV pseudoviruses. (B to D) SPR-measured affinity for SUDV GP of the parental antibody and the 2 triple mutants. (E to H) Structural simulation of key residue changes (H27Q, S52Y, G66R; A50Y, S52Y, L54R) at the antibody–antigen interface.

All variants were screened at 10 μg/ml against SUDV pseudovirus. Wild-type ADI-15946 showed about 70% neutralization at this concentration. Among the tested variants, 25 exhibited improved neutralization (Fig. 4K), with multi-point mutants showing the greatest enhancement (Fig. 4L and Table 4). Secondary screening against EBOV and BDBV pseudoviruses confirmed specificity (Fig. 4M). Notably, within this constrained combinational design space, the triple mutants H27Q/S52Y/G66R-LC and A50Y/S52Y/L54R-LC emerged as top-performing variants, enhancing SUDV neutralization by >40-fold and >100-fold, respectively, without compromising activity against EBOV or BDBV (Fig. 5A).

**Table 4:** ADI-15946 mutations affect neutralizing activity against 3 Ebola viruses

	EBOV				BDBV				SUDV			
	IC_90_ (μg/ml) ^a^	Fold change	IC_50_ (μg/ml) ^b^	Fold change ^c^	IC_90_ (μg/ml)	Fold change	IC_50_ (μg/ml)	Fold change	IC_90_ (μg/ml)	Fold change	IC_50_ (μg/ml)	Fold change
H27Q/S52Y/G66R-LC	0.86	1.28	0.15	1.09	0.40	0.52	0.05	0.58	0.69		0.08	121.17
T32W/S52Y/L54R-LC	1.87	0.59	0.33	0.51	1.09	0.19	0.24	0.12	/	/	2.51	3.65
A50Y/S52Y/L54R-LC	1.10	1.00	0.15	1.12	0.20	1.06	0.03	1.08	10.55		0.22	40.94
I29Y/A50Y/S52Y-LC	7.00	0.16	1.10	0.15	1.77	0.12	0.46	0.06	/	/	0.48	19.23
I48L-LC	0.47	2.34	0.06	2.82	0.11	1.97	0.02	1.52	380.35		3.02	3.03
A51M-LC	2.11	0.52	0.37	0.45	0.51	0.41	0.07	0.41	/	/	0.62	14.75
L54P-LC	2.23	0.50	0.46	0.36	0.20	1.05	0.05	0.55	/	/	1.97	4.65
E46L-HC	1.42	0.78	0.21	0.79	0.19	1.11	0.02	1.17	1030.39		1.08	8.52
E46I-HC	1.75	0.63	0.40	0.42	0.16	1.28	0.02	1.20	/	/	1.82	5.03
V48C-HC	1.48	0.75	0.24	0.70	0.24	0.87	0.04	0.81	/	/	0.97	9.49
ADI-15946	1.11	1.00	0.17	1.00	0.21	1.00	0.03	1.00	/	/	9.16	1.00

^a^ IC_50_ denotes the antibody concentration required to inhibit 50% of the luciferase signal expression following pseudovirus infection (μg/ml).

^b^ IC_90_ denotes the antibody concentration required to inhibit 90% of the luciferase signal expression following pseudovirus infection (μg/ml).

^c^ Fold change denotes the multiple by which ADI-15946’s IC_50_ exceeds that of the mutant antibody, or by which ADI-15946’s IC_90_ exceeds that of the mutant antibody.

Since ADI-15946 targets the monomeric GP protein, it can be directly used for affinity testing with commercially available GP. The affinity of ADI-15946 for SUDV-GP is 2.241 nM (Fig. 5B). Results from single-point mutagenesis indicate that the region near position 52 of the light chain plays a key role in enhancing neutralizing activity against SUDV (Fig. 4K). The S52Y mutation is present in both 3-point mutants, and the introduction of a benzene ring in the side chain directly improves antigen-binding capability.

Our structure analysis reveals that the key mutations establish a complementary network of specific, directional noncovalent interactions that optimize the antigen-binding interface. For instance, the L54R mutation converts a weak, nonspecific hydrophobic interaction (3.5 Å) into a cation–π interaction, where the guanidinium group of arginine forms an electrostatic contact with the aromatic ring of the antigen, shortening the interaction distance to approximately 2 Å. Simultaneously, the S52Y substitution introduces a π–π stacking interaction (~4.5 Å, oblique orientation) that further stabilizes the complex (Fig. S15).

Together, the newly introduced tyrosine and arginine residues form a complementary interaction network that enhances binding free energy and reduces conformational entropy loss by improving interface complementarity and rigidity. As a result, the cooperative effect of these mutations exceeds the simple sum of individual interaction energies, leading to a marked improvement in antibody affinity and neutralizing potency.

The H27Q/S52Y/G66R-LC variant incorporates single-point mutations within the light chain’s CDR1, CDR2, and FR3 regions. SPR experiments showed that this antibody binds to SUDV-GP with an affinity of 0.349 nM (Fig. 5C and Table S2), representing a 6.4-fold improvement compared to ADI-15946. These combined mutations increased the antigen–antibody contact area from 482.46 Å^2^ to 569.35 Å^2^ (Fig. 5E and F), enhancing interactions with residues A526, A527, and G528 on GP2. The A50Y/S52Y/L54R-LC variant, which contains 3-point mutations in the light chain CDR2 region, exhibited an affinity of 0.248 nM for SUDV-GP (Fig. 5D and Table S1), corresponding to a 9-fold enhancement over ADI-15946. This mutation enlarged the interaction area between GP and the light chain CDR2 loop from 62.59 Å^2^ to 215.23 Å^2^ (Fig. 5G and H), which explains the substantial gain in neutralization potency.

The interaction surface area between the mutant antibodies and the EBOV-GP protein increased, which may help the antibodies maintain their original neutralizing activity against EBOV (Fig. S16). Previous research has shown that ADI-15946, when combined with the TIM-1 extracellular domain (ECD) proteins, exhibits synergistic inhibitory effects on EBOV entry [29]. Our docking results further revealed that the binding of ADI-15946 to SUDV-GP enlarges the interaction surface of the GP protein region encompassing residues A530 to H549. Notably, T544 within this region is a key residue for TIM-1 binding. The introduced mutations enhanced the antibody’s affinity for this specific region. In competitive binding assays, the mutant antibodies demonstrated a higher affinity for GP than TIM-1, correlating with their increased neutralizing activity against SUDV (Fig. 5F and H).

## Discussion

This study establishes and validates a computational–experimental pipeline for rapidly optimizing therapeutic antibodies against highly mutable viruses such as Ebola. The approach centers on a structure-guided closed-loop framework that begins with antigen–antibody complexes, utilizes computational analysis and mutation scanning, and iteratively refines designs through parallel wet-lab validation across viral strains. This systematic process enables the evolution of antibodies toward enhanced breadth and potency.

Our design strategy focused on the conserved IFL epitope. Sequence alignment across 6 Ebola species confirms that residues critical for binding by antibodies ADI-15878 and ADI-15946 are largely invariant (Fig. S17). Nonconserved positions predominantly involve amino acids with similar physicochemical properties, and key interaction surfaces strengthened through engineering, such as the contact with glycosylation site N204 in variant W32G-LC, are also conserved. This evolutionary constraint provides a solid foundation for achieving broad-spectrum neutralization.

The pipeline yielded 2 principal advances. First, FR–CDR co-engineering of ADI-15878 produced variant W32G-LC, which increased neutralization against EBOV (17-fold), BDBV (7-fold), and SUDV (2-fold) while maintaining structural integrity (Fig. 3D and Fig. S1). Second, for ADI-15946, a docking-guided deep mutation-scanning strategy informed by a multi-template consensus approach generated variants such as H27Q/S52Y/G66R-LC and A50Y/S52Y/L54R-LC. These exhibited dramatic neutralization improvements against SUDV (>40-fold and >100-fold) without compromising activity against EBOV or BDBV. These outcomes underscore the importance of evaluating combinatorial mutations to avoid the antagonistic effects often seen in single-point mutagenesis, thereby preventing over-optimization toward a single strain.

Our analysis further revealed a conditional, context-dependent correlation between computational predictions and experimental neutralizing potency. Although no linear correlation was found between predicted ΔΔG_bind_ and changes in IC_50_, sensitivity analysis confirmed that the enrichment of experimentally validated mutations remained robust across moderate variations in parameter thresholds, underscoring the stability of our selection criteria. This finding directly informed our refined screening strategy. While initial single-point scanning of ADI-15878 yielded a positive hit rate of 23.5%, we adopted a more data-driven approach for ADI-15946. First, we used single-mutation neutralization data to select optimal structural templates and define consensus selection criteria, prioritizing interface residues (BSA > 0) with favorable ΔΔG_bind_ and RSA% profiles. This strategy increased the success rate to 32.8% for single mutations and further to 62.5% for multi-point combinations. Unlike previous AI-driven designs that primarily relied on binding affinity for validation, our study implements a closed-loop optimization framework. This framework iteratively couples computational prediction with neutralization assays. Predictions guide experimental screening, and experimental results in turn refine the computational models and selection thresholds, enabling synergistic gains in both antibody breadth and potency. This iterative, experiment-guided workflow not only enhances design efficiency but also establishes a generalizable and optimizable strategy for developing broad-spectrum neutralizing antibodies against highly variable pathogens.

Several limitations remain. First, dependence on existing PDB or docking models may introduce template bias, particularly for antigenically variable viruses; future work could incorporate AI-predicted antigen structures, such as those from AF3 [30,31], to broaden coverage. Second, while pseudovirus assays validated our designs, in vivo studies are necessary to confirm therapeutic efficacy. Third, the rapid evolution of RNA viruses requires continuous pipeline updates to address emerging escape variants. Despite these challenges, the combined use of docking, multi-parameter modeling, and focused wet-lab screening offers a rapid and scalable strategy for developing broad-spectrum antibodies. The success of multi-point mutants demonstrates that systematic interface optimization outperforms traditional single-point mutagenesis. This approach can be extended to other pathogens where balancing potency and breadth remains challenging, such as influenza or severe acute respiratory syndrome coronavirus 2 (SARS-CoV-2), and may also aid in optimizing antibody-based detection methods by improving the sensitivity of probe antibodies [32].

In summary, our work establishes a model-driven, closed-loop optimization strategy for broad-spectrum antibody design. Key transferable principles include FR–CDR co-engineering to remodel the paratope scaffold, multidimensional parallel screening to quantitatively balance potency across strains, and multi-template consensus assessment to reduce model bias and enhance robustness, particularly in the absence of high-resolution structures. All selection rules were defined a priori, ensuring a generalizable computational framework that extends beyond individual antibody–antigen pairs. Together, these results demonstrate how integrated computational design can systematically improve both antibody breadth and potency.

## Materials and Methods

### Study design

#### Research objective

This study aims to computationally analyze random amino acid mutations in antibodies to identify variants with enhanced broad-spectrum neutralizing activity against EBOVs.

#### Research subjects or units

The optimized antibodies ADI-15878 and ADI-15946 are both EBOV neutralizing antibodies, whose sequences are available from the PDB database.

#### Experimental design

We combined computational simulations with directed wet-lab experiments to screen for variants exhibiting enhanced broad-spectrum neutralization capacity. When complex structures were available, PDB complex structures served as analysis templates; when complex structures were absent, complex structures were generated via molecular docking and used as analysis templates. Potential mutations identified through computational analysis undergo multiple rounds of pseudovirus neutralization screening. By comparing neutralization assay results with computational predictions, high-quality docking templates are selected for multi-site mutagenesis. Pseudovirus neutralization assays are then performed to evaluate the impact of these mutations on neutralizing activity and broad-spectrum efficacy. Computationally identified potential mutations underwent multiple rounds of pseudovirus neutralization screening to evaluate their impact on neutralizing activity and broad-spectrum efficacy. Enhanced antibody binding affinity was ultimately validated using flow cytometry (FACS) or SPR techniques.

#### Sample size

Antibody single-point or multi-point mutations were predicted via computer scanning, selecting sites with ΔΔG_bind_ increases exceeding 1 for testing. But due to alignment-induced bias in the SUDV–GP–ADI-15946 docking model, the selection range was expanded, adopting ΔΔG_bind_ > 0.2 as the criterion.

#### Outliers

Mutant antibodies were tested against the prototype antibody in the same pseudovirus neutralization system. Antibodies showing enhanced neutralization activity were identified using the prototype antibody’s IC_90_ and IC_50_ values as thresholds. After screening with multiple Ebola pseudoviruses, antibodies demonstrating broad-spectrum neutralization enhancement were ultimately selected.

In binding capacity comparison experiments, the prototype antibody’s binding affinity served as the baseline for comparison.

#### Replicates

Data structures were validated through 3 or more neutralization assays. The optimized antibodies ultimately obtained were verified as effective through protein purification and neutralization assays across different batches.

#### Blinding

All antibodies in the experiments were assigned random sequential codes; mutation sites were not directly labeled.

### Computational mutation scanning

This study established an iterative optimization workflow that integrates computational prediction with experimental validation to enhance the breadth and potency of antibody neutralization (Fig. 1B). Methodologically, the workflow began with the use of PDB structures (e.g., 6DZL and 6MAM) or the generation of complex models through constrained molecular docking for interface analysis. Subsequently, systematic saturation mutagenesis was performed on all CDR residues and neighboring residues within 4 Å, evaluating all 20 possible amino acid substitutions. This was carried out using mCSM-AB [33] and mmCSM-AB [34] algorithms to predict changes in binding free energy (ΔΔG_bind_) and relative solvent accessibility (RSA%). The results generated a ranked list of variants based on these parameters. In cases where model uncertainty was high, such as during the optimization of ADI-15946 against SUDV, screening thresholds were dynamically adjusted. Specifically, the RSA% cutoff was relaxed from a minimum of 10 to 0, and ΔΔG_bind_ > 1. This was complemented by applying a “ten-model consensus trend” criterion to improve prediction reliability (Figs. S11 to S14). Heatmaps were used to visualize predicted affinity changes, and candidates exhibiting high predicted ΔΔG_bind_ and favorable RSA% values were prioritized for further experimental validation.

### FR optimization and CDR grafting

When direct interface optimization failed to improve both breadth and potency, we turned to FR grafting to subtly modulate CDR conformation. To identify human antibody FRs structurally compatible with the parental ADI-15878 scaffold, we performed a structural similarity search using Foldseek [35]. The parent antibody PDB file was uploaded to the Foldseek web server (https://search.foldseek.com/) with the following settings: database PDB100 (20240101), mode “3Di/AA”, and iterative search disabled. From the search results for the light and heavy chains, only structures derived from *Homo sapiens* were considered. Candidate selection was primarily based on 2 alignment metrics provided by Foldseek: TM-score and RMSD. Higher TM-scores (closer to 1) and lower RMSD values indicated better global structural conservation. These results were further validated through manual visual inspection of the superposed structures. Importantly, candidate FRs were selected only if the same donor PDB file yielded well-aligned structures for both the light chain and the heavy chain. Typically, we selected the top 3 to 5 PDB files with the highest alignment scores as donor frameworks for grafting. The selected FRs were then grafted onto the parental antibody according to the international ImMunoGeneTics information system (IMGT) numbering, and the resulting models were assessed to ensure overall structural integrity before proceeding to experimental validation.

### Evaluation of antibody humanity

To evaluate the degree of antibody humanization in this study, we employed AbNativ, a deep-learning tool designed to assess antibody “nativeness”. This tool analyzes the characteristics of input antibody sequences and systematically compares them against a specialized database of natural human antibody sequences. Based on this comparison, AbNativ assigns a quantitative score reflecting the degree of similarity between the designed antibody and natural human antibodies, thereby providing a measurable indicator of humanization level.

### Docking and molecular dynamics

When experimental complex structures were unavailable or incomplete, we performed constrained docking to generate antibody–GP models. The BSA interaction sites between ADI-15946 and EBOV GP in the PDB structure 6MAM were analyzed. The structures of ADI-15946 and the GP protein monomer were then separately input into docking software. Specifically, during the docking process (e.g., ZDOCK), residues within 4 Å of the known interface were selected as constraints, and erroneous conformations hindering GP trimerization were excluded to ensure model reliability. BSA > 0 interaction sites on both molecules, along with nonbinding regions, were constrained during docking. Ten docking models were generated. These models were subsequently superimposed onto the GP trimer in 6MAM to identify the most structurally compatible complex, which was considered the correct docking model.

The EBOV–GP–ADI-15946 docking models were imported into the iMODS platform [36,37] for feasible transition pathways analysis. Using the docking model as the initial model and the 6MAM file from the PDB database as the target model, we performed molecular dynamics simulations to obtain the simulated model ultimately.

### AlphaFold 3 prediction

First, the PDB:6DZL file is divided into 3 sections: sequence 1: GP protein amino acid sequence (GP1 + GP2), sequence 2: ADI-15878 light chain amino acid sequence, and sequence 3: ADI-15878 heavy chain amino acid sequence. For sequence 1, the copy parameter is set to 3, while for the other 2 sequences, the copy parameter is set to 1 for prediction. Subsequent predictions involved altering the amino acid sequences of the antibody’s light and heavy chains to generate mutant antibody–antigen complexes. Additionally, the PDB:6MAM file was divided into 3 parts: sequence 1: GP protein amino acid sequence (GP1 + GP2), sequence 2: ADI-15946 light chain amino acid sequence, and sequence 3: ADI-15946 heavy chain amino acid sequence. The parameter settings remain unchanged from before.

### Determination and analysis of BSA

Using the PDBePISA website, we imported the PDB file into the webpage. We set the Processing mode to auto, selected interfaces, and obtained the BSA analysis for each amino acid in the complex. This study employs PyMOL software combined with surface area analysis to quantitatively measure protein–protein interactions. The specific operational steps are as follows: load the PDB files for each chain of antigen A and antibody B separately, merge them to construct the complex, and calculate the interface area using the following formula: “Surface area of monomer A + Surface area of monomer B − Surface area of the complex”. A is the PDB file for EBOV GP or SUDV GP, B is the PDB file for ADI-15878 or ADI-15878 light chain CDR1, or ADI-15946 or ADI-15946 light chain CDR2 region.

### Cell culture

293T cells and Huh7 cells were cultured in Gibco Dulbecco’s modified Eagle’s medium (Gibco DMEM; ThermoFisher Scientific, C11995500BT) supplemented with 10% fetal bovine serum (Gibco FBS; ThermoFisher Scientific, 10099-141) and 1% penicillin–streptomycin (Solarbio, P1400) at 5% CO_2_, 37 °C. 293F cells were cultured in SMM 293-TII Expression Medium (serum-free, complete medium, Sino Biological) at 5% CO_2_, 37 °C, 125 rpm.

### Antibody plasmid point mutation

Based on the results of mutation analysis, the sites for antibody mutation were selected (Mut Express II Fast Mutagenesis Kit, Vazyme, C214-01). Specific primers containing mutation sites were designed based on the target site. Using the wild-type plasmid as the template, polymerase chain reaction (PCR) amplification was performed using high-fidelity DNA polymerase. The amplified products were digested with the Dpn I enzyme at 37 °C for 1 h to remove the template plasmid, and then transformed into DH5α (TransGen, CD201). Positive clones were picked and sent for sequencing to verify the mutation sites.

### Monoclonal antibody production and purification‌

All monoclonal antibodies (mAbs) were produced by transient transfection in Expi293F cells, using the pFuse-hIgG1-Fc1 vector system (Fig. S18). Genes encoding antibody heavy and light chains were cloned into the pFuse-hIgG1-Fc1 vector. Heavy- and light chain sequences were cloned into the Eco RI and Bgl II sites, respectively, and expressed as full-length human immunoglobulin G1 (IgG1) without any extra purification or detection tags to avoid influencing native binding properties. Proteins were produced by transient transfection of 293F cells following the manufacturer’s protocol and purified from filtered culture supernatants using Protein A MagBeads MX (GenScript, L00672-4), with typical yields of 8 to 12 mg per liter of culture. The bound antibodies were washed with phosphate-buffered saline (PBS) adding 0.1% Tween, eluted with 200 mM acetic acid (pH 3.5), 50 mM NaCl into one-eighth volume 2 M Hepes (pH 8.0), and buffer-exchanged into PBS, filtered using sterile 0.22-μm pore size filter devices (Millipore, SLHP033RS), concentrated, and stored in aliquots at −80 °C until use. Size exclusion chromatography confirmed >95% monomeric purity for all variants (Fig. S19).

### Prepare EBOV GP pseudotyped particle

GP gene sequences from EBOV (GenBank: AF086833.2), Bundibugyo virus (BDBV; GenBank: MK028856.1), and Sudan virus (SUDV; GenBank: NC_006432.1) were cloned individually into the pcDNA3.1 vector. HEK293T cells were seeded at 2.5 × 10^6^ cells per 10-cm dish 1 d before transfection. Cells were transfected using PEI (Polysciences Inc., 24765-100) with a mixture containing 10 μg of pNL4.3-Luc-E−R− [38] and 10 μg of GP-encoding pcDNA3.1 plasmid per dish in 1-ml total volume. A non-enveloped lentivirus particle (bald virus) served as a negative control. Sixteen hours post-transfection, the media were replaced with fresh media containing 2% FBS. Supernatants containing EBOV GP pseudotyped particles were harvested 36 to 48 h post-transfection, filtered through a 0.22-μm syringe filter to remove debris, and either used fresh or aliquoted and stored at −80 °C.

### Neutralization assays

Huh7 cells were seeded in 96-well plates 1 d before transduction. Pseudovirus particles were added in the presence or absence of serially diluted mAb. Forty-eight hours post-transduction, cells were lysed using 50 μl of passive lysis buffer. Luciferase activity was measured by incubating 50 μl of lysate with 50 μl of luciferase assay substrate (Promega, E1501) according to the manufacturer’s instructions [39,40]. All studies with infectious pseudotyped particles were performed under biosafety level 2 (BSL-2) conditions at the Chinese Academy of Medical Sciences, the Institute of Pathogen Biology.

### Flow cytometry analysis (FACS)

293T cells were transfected with pcDNA3.1 vectors encoding either wild-type EBOV-GP or the EBOV-GP G528E mutant. Twenty-four hours post-transfection, cells were harvested, washed 3 times with PBS (Solarbio, P1010), and fixed with 4% paraformaldehyde (Solarbio, P1110). Fixed cells were incubated with ADI-15878 and W32G-LC antibodies at concentrations ranging from 50 μg/ml to 0.016 μg/ml as a 5-fold serial dilution for 1 h at room temperature. Cells were then washed 3 times with PBS and stained with allophycocyanin (APC)-conjugated anti-human IgG antibody (Jackson ImmunoResearch, 709-136-098) for 30 min at room temperature. After 3 final PBS washes, samples were analyzed by flow cytometry.

### SPR assay

SPR experiments were conducted at room temperature using a BiaCore T200 with CM5 sensor chips (GE Healthcare). Both the sample and reference flow cell surfaces were activated with a 1:1 mixture of 0.1 M NHS (N-hydroxysuccinimide) and 0.1 M EDC [3-(N, N-dimethylamino) propyl-N-ethylcarbodiimide] at a flow rate of 10 μl/min. The reference flow cell remained unmodified for comparison. All surfaces were blocked with 1 M ethanolamine (pH 8.0). The running buffer was HBS-EP (0.01 M Hepes, pH 7.4; 150 mM NaCl; 3 mM EDTA; 0.05% surfactant P-20). The antigen was diluted in 10 mM acetate buffer (Cytiva, pH 5.5, BR100352) and covalently immobilized on the sensor chip via amine coupling (Cytiva Amine Coupling Kit, BR100050). After achieving a coupling density of 600 response units (RU), excess antigen was removed by washing. Remaining reactive sites were blocked with ethanolamine. Antibodies were serially diluted 2-fold in running buffer across a concentration range of 0.25 to 4 nM. After each cycle, the sensor surface was regenerated with 10 mM glycine–HCl at pH 2.5. All procedures followed the manufacturer’s recommended protocols for protein affinity analysis (Biacore Systems, Cytiva).

### Statistical analysis

Neutralization IC₅₀ and IC₉₀ values were calculated from antibody titration curves using 4-parameter nonlinear regression analysis in GraphPad Prism 5 software.

## Data Availability

All software tools and platforms used here are publicly available. Further information and requests for resources and reagents should be directed to and will be fulfilled by the lead contact, W.Y. (wyang@ipb.pumc.edu.cn).
