# Supplementary material for: Computational Design of Broad-Spectrum Ebola Antibodies through Framework and Complementarity-Determining Region Synergistic Optimization
Source: Research (Wash D C). 2026 Mar 23;9:1211. doi: 10.34133/research.1211 (PMC13006735; doi:10.34133/research.1211)
Supplement: Supplementary 1 — Figs. S1 to S19 Tables S1 and S2 [file research.1211.f1.docx]

**SUPPLEMENTARY MATERIALS**

**Computational Design of Broad-Spectrum Ebola Antibodies through Framework and Complementarity-Determining Regions Synergistic Optimization**

Xinhui Zhang^a, b^, Xiuying Liu^a, b^, Jingya Zhou^a, b^, Shengnan Pan^a, b^, Xuehua Yang^a, b^, Huarui Duan^a, b^, Yi Liao^a, b^, Peixiang Gao^a, b^, Fangyuan Zhang^a, b^, Xuemeng Dong^a, b^, Junyu Liu^a, b^, Xiaojing Chi^a, b, *^ Wei Yang^a, b, c, *^

**Contents**

Supplementary Figure S1 to S19: Page 2 to21

Supplementary Table S1 to S2: Page 22 to 23

**Fig. S1**


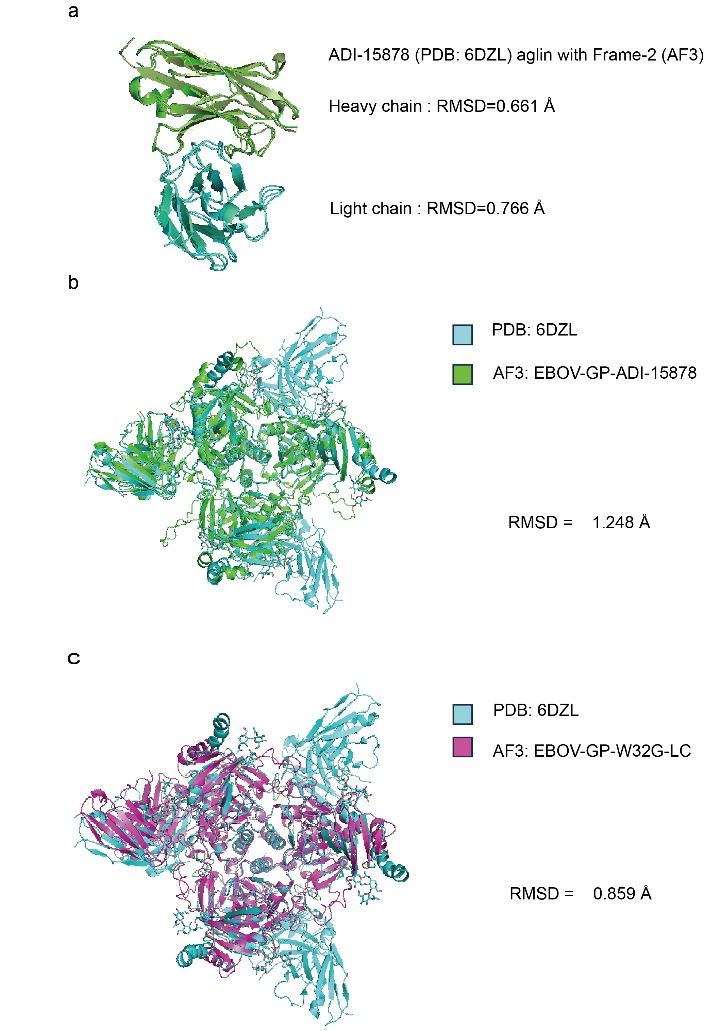


***Fig. S1.*** *AF3 prediction Frame-2, EBOV-GP-ADI-15878, EBOV-GP-W32G-LC compared with file 6DZL in the PDB database.* *(a) AF3 prediction of the Frame-2 structure was compared with ADI-15878 (PDB: 6DZL). (b) The ADI-15878 and EBOV-GP protein amino acid sequences were imported into AF3 for structural and docking predictions. The predicted results were compared with 6DZL in the PDB database for similarity, yielding an RMSD value of 1.248 Å. (c) The W32G-LC and EBOV-GP protein amino acid sequences were imported into AF3 for structural and docking predictions. The predicted results were compared with 6DZL in the PDB database for similarity, yielding an RMSD value of 0.859 Å.*

**Fig. S2.**

***
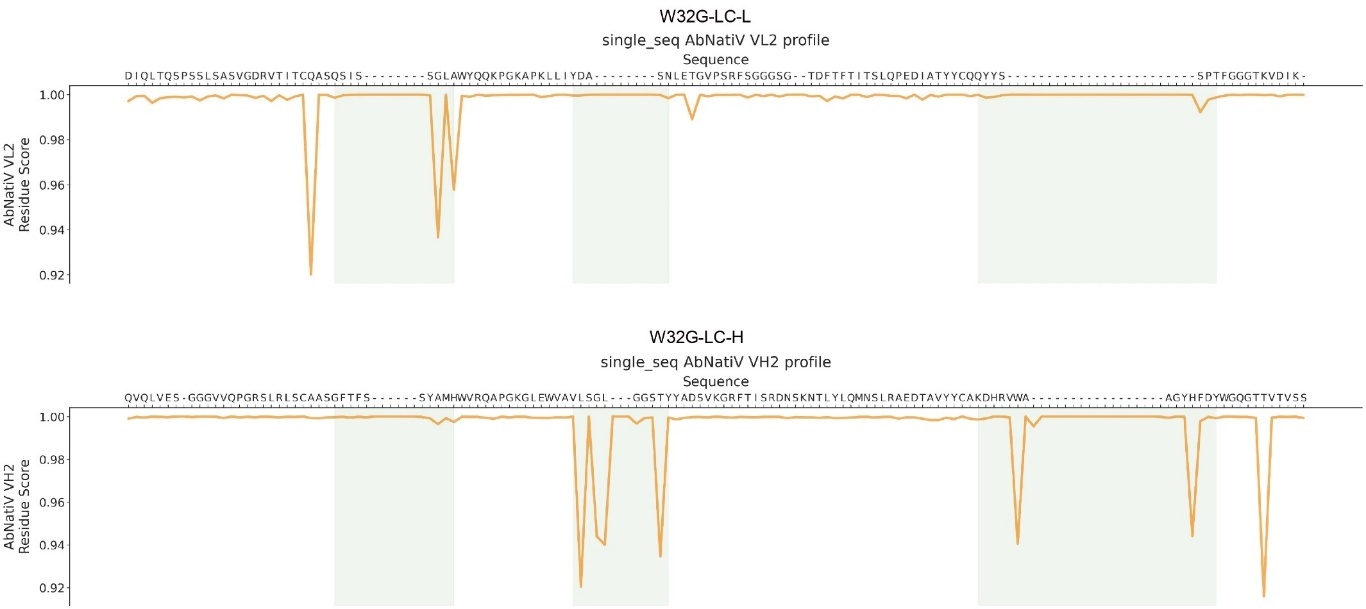
***

***Fig. S2.*** *Assessment of the human nativeness of the optimized antibody light and heavy chains. The shaded areas respectively represent the six CDR regions, while the other parts represent the framework regions.*

**Fig. S3.**


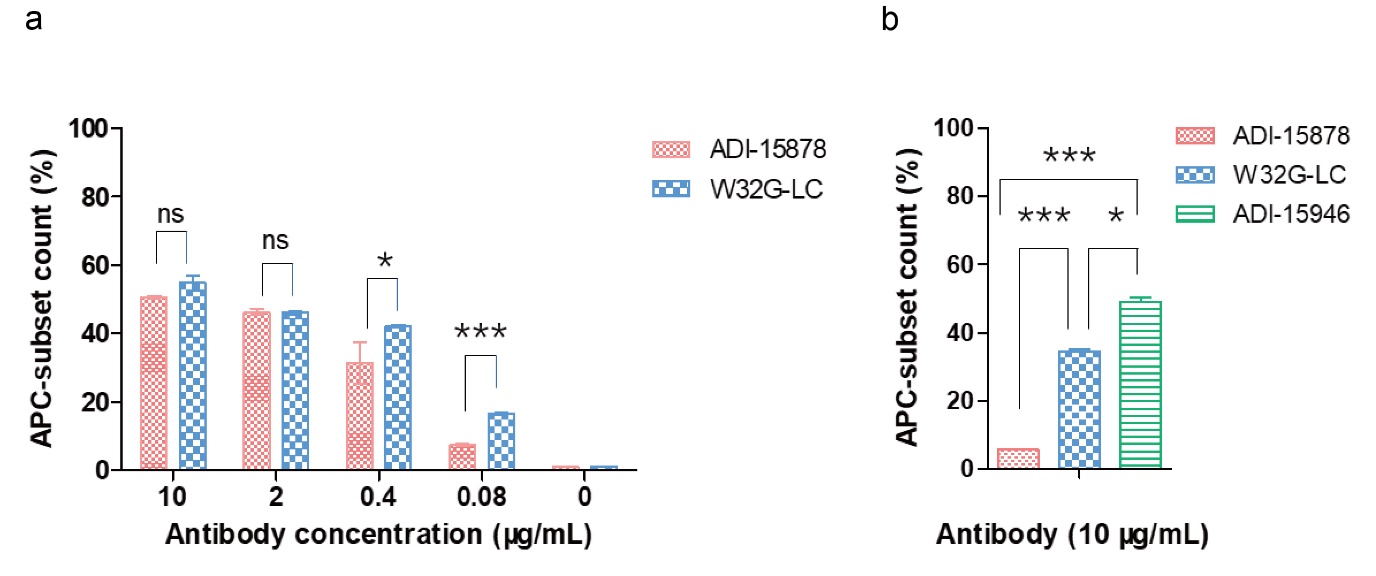


***Fig. S3****. ADI-15878 and W32G-LC demonstrate differences in GP protein binding capacity in streaming mode. (a) Detection of EBOV-GP Protein Concentration Gradient Dependence Using ADI-15878 and W32G-LC. (b) W32G-LC enhances the binding affinity of ADI-15878 to EBOV-GP G528E, with ADI-15946 serving as the positive control antibody.*

**Fig. S4.**

**
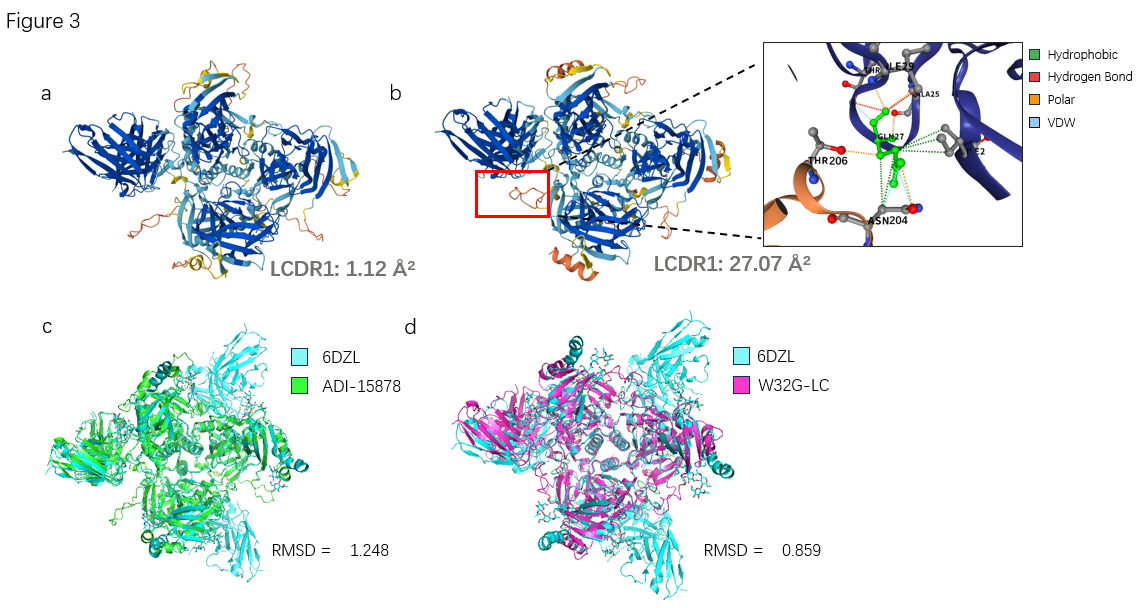
**

***Fig. S4.*** *Structural mechanisms underlying the improved performance of W32G-LC. (a) AF3-predicted structure of the ADI-15878 antibody in complex with the EBOV GP, showing high consistency with the experimental model (PDB 6DZL). (b) AF3- predicted structure of the W32G-LC and EBOV complex, revealing an additional binding epitope involving residues N204 and T206 compared with ADI-15878. (c) Structural alignment between the AF3-predicted ADI-15878–GP complex and the corresponding PDB reference mode. (d) Structural alignment between the AF3-predicted W32G-LC–GP complex and the corresponding PDB reference model, demonstrating consistent binding orientation.*

**Fig. S5.**

**
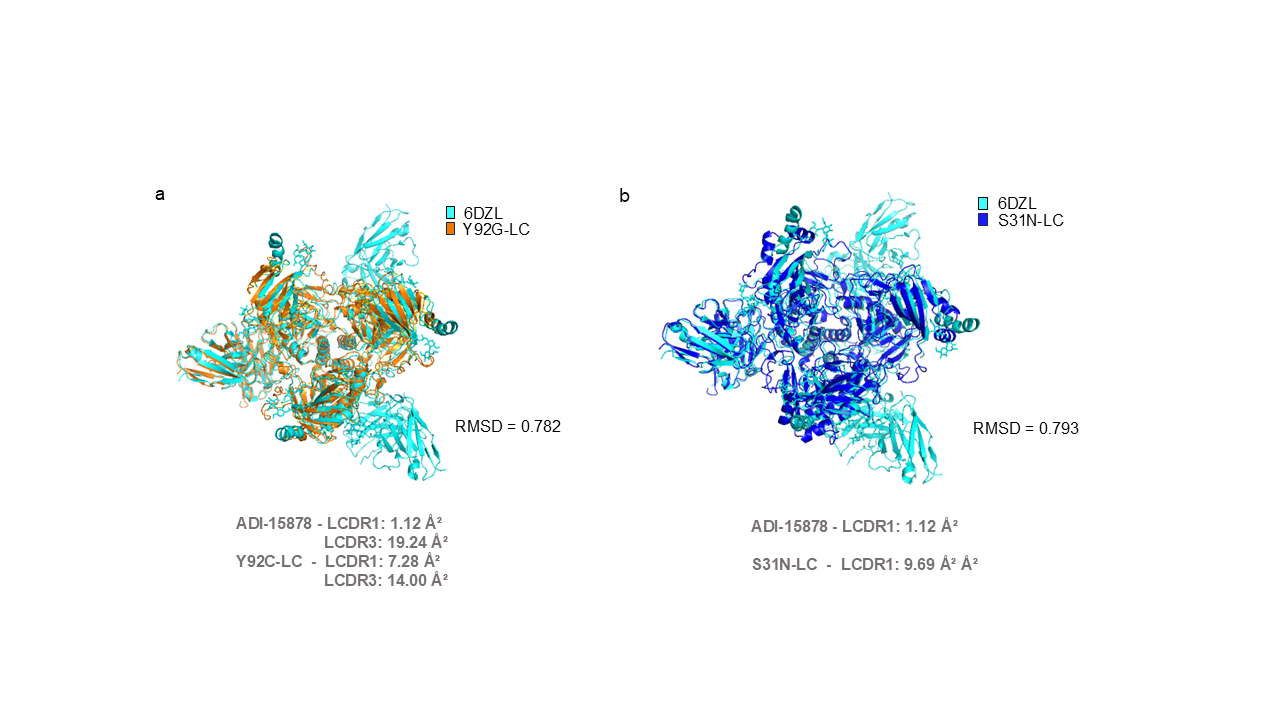
**

***Fig. S5.*** *Prediction of other point mutation complexes and BSA changes. (a) Structural alignment between the AF3-predicted Y92G-LC–GP complex and the corresponding PDB reference mode. The BSA change in the CDR region where the point mutation occurs. (b) Structural alignment between the AF3-predicted S31N-LC–GP complex and the corresponding PDB reference model, demonstrating consistent binding orientation The BSA change in the CDR region where the point mutation occurs.*

**Fig. S6.**

**
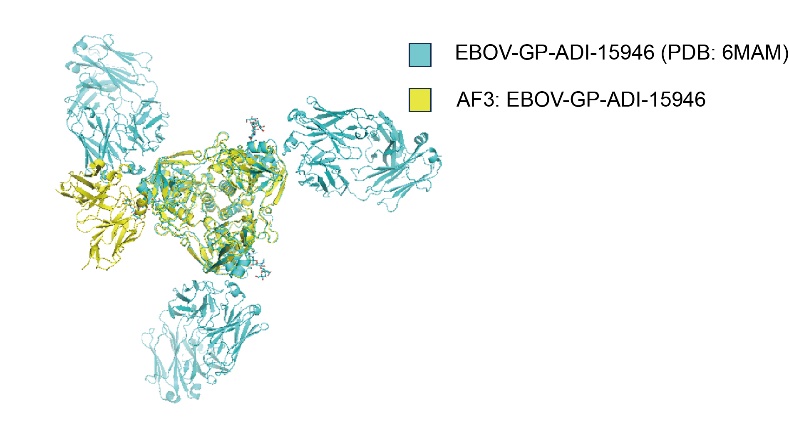
**

***Fig. S6.*** *AF3 prediction EBOV-GP-ADI-15946 compared with file 6MAM in the PDB database. The ADI-15946 and EBOV-GP protein amino acid sequences were imported into AF3 for structural and docking predictions. The predicted results were compared with 6MAM in the PDB database for similarity.*

**Fig. S7.**

**
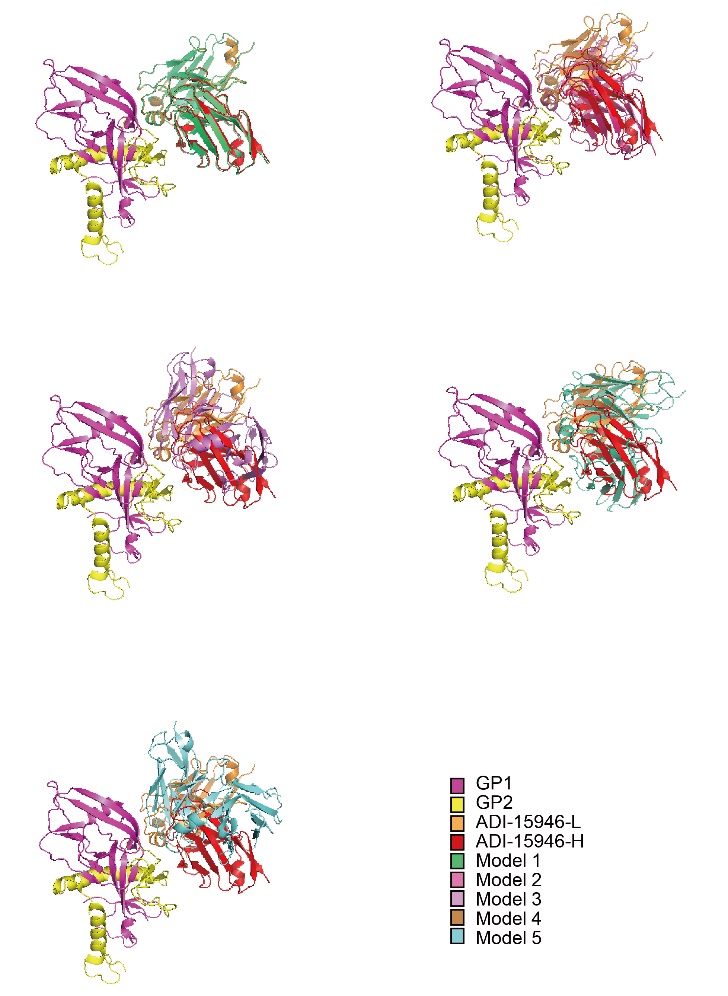
**

**Fig. S7.** *Molecular docking (HDOCK) ADI-15946 with EBOV-GP.*

**Fig. S8.**

*
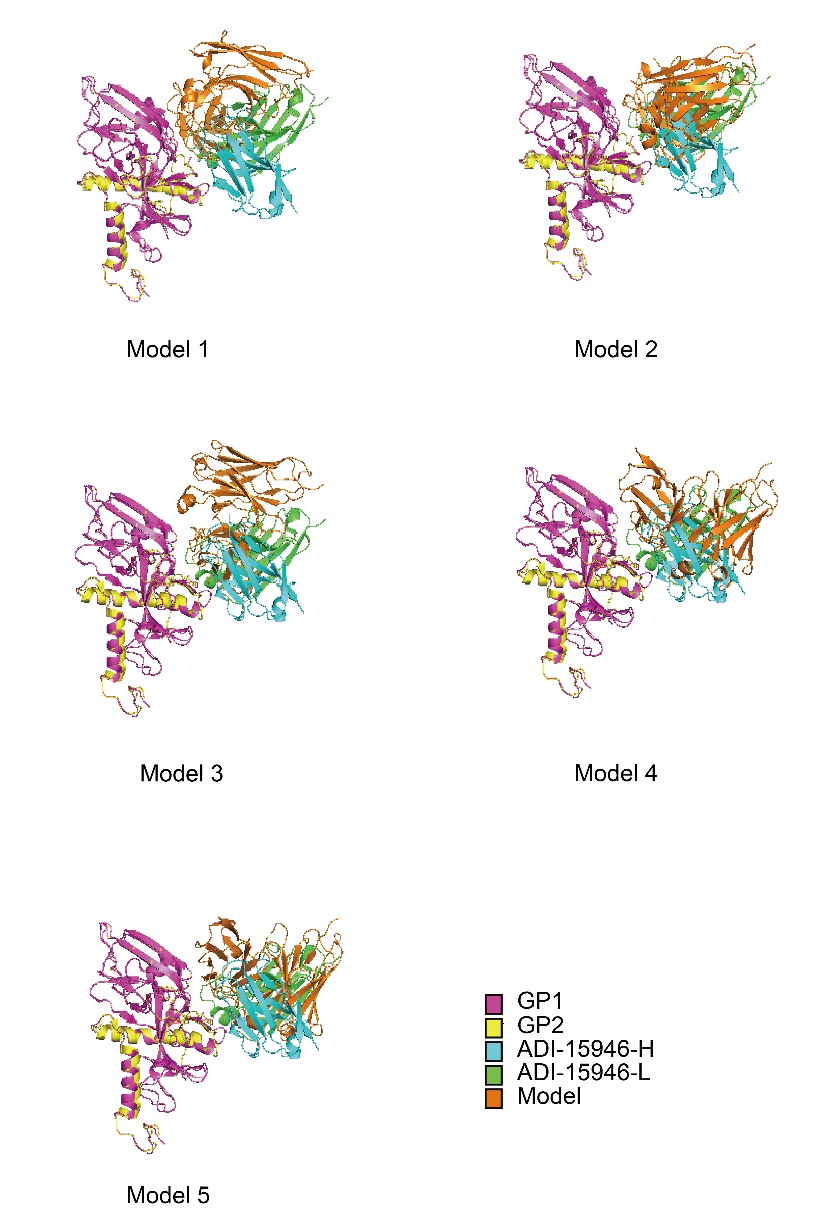
*

**Fig. S8.** *Molecular docking (HDOCK) ADI-15946 with SUDV-GP.*

**Fig. S9.**


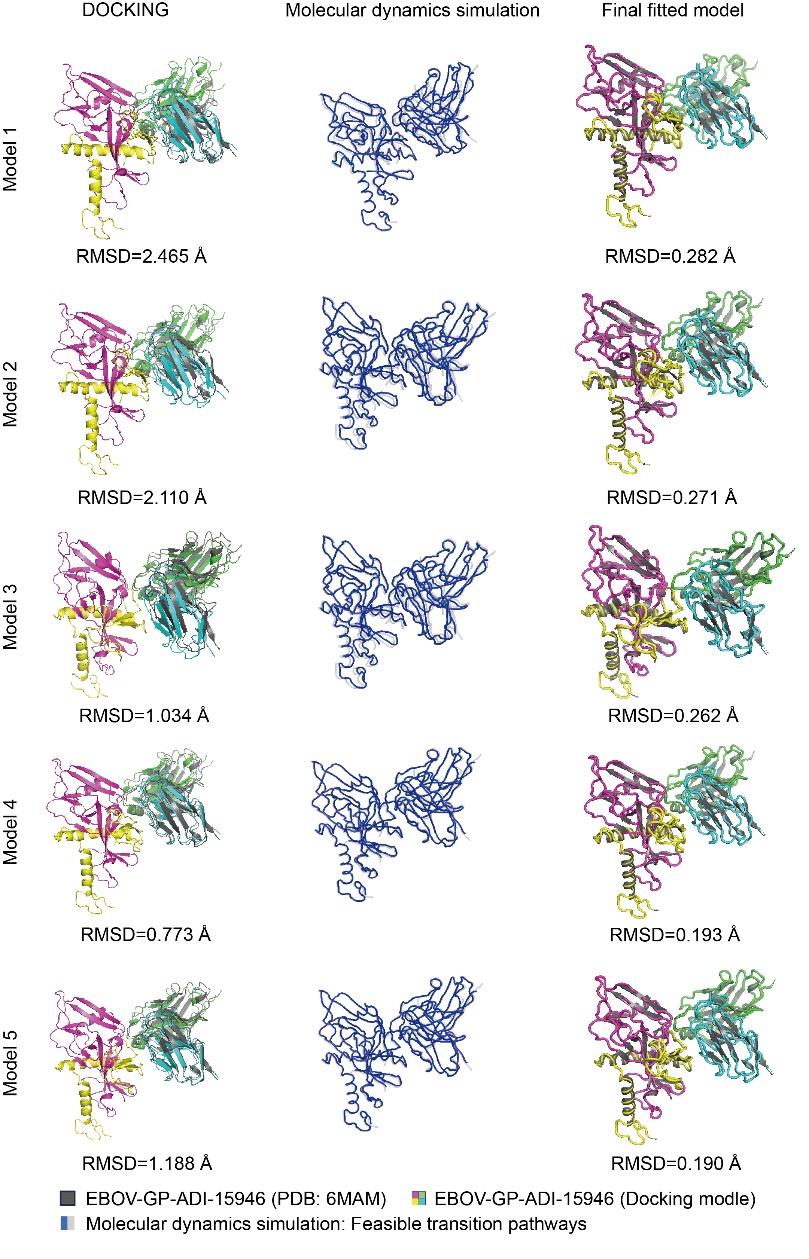


***Fig. S9.*** *Molecular docking and molecular dynamics simulation evaluation of ADI-15946 with EBOV-GP. Molecular dynamics simulations were performed on the five models obtained from the molecular docking between EBOV-GP and ADI-15946. The final fitted model underwent similarity analysis with the 6MAM file in the PDB database. The final RMSD value is within 0.3 Å.*

**Fig. S10.**

**
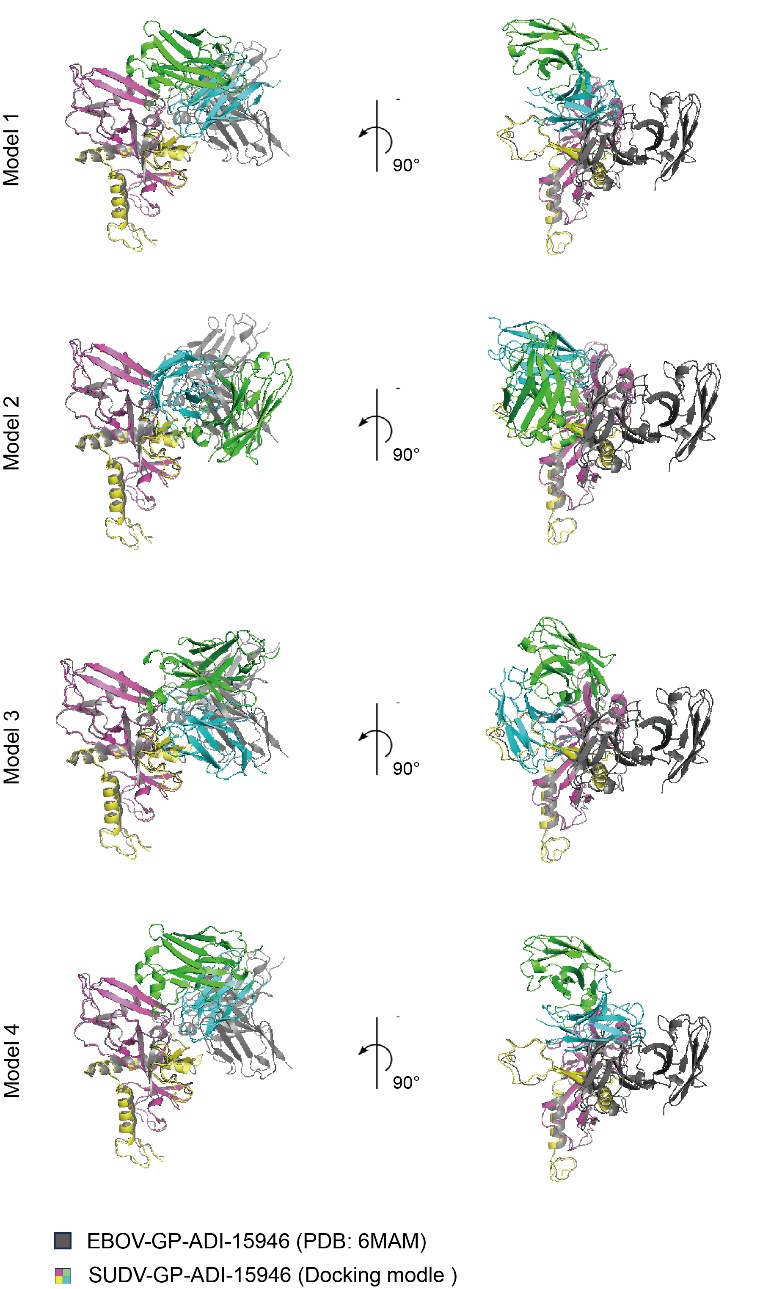
**

***Fig. S10.*** *Molecular docking model of SUDV-GP with ADI-15946. Using the same method, we generated docking models for ADI-15946 and SUDV-GP proteins. We observed a shift in the binding site compared to ADI-15946's interaction with EBOV-GP.*

**Fig. S11.**

**
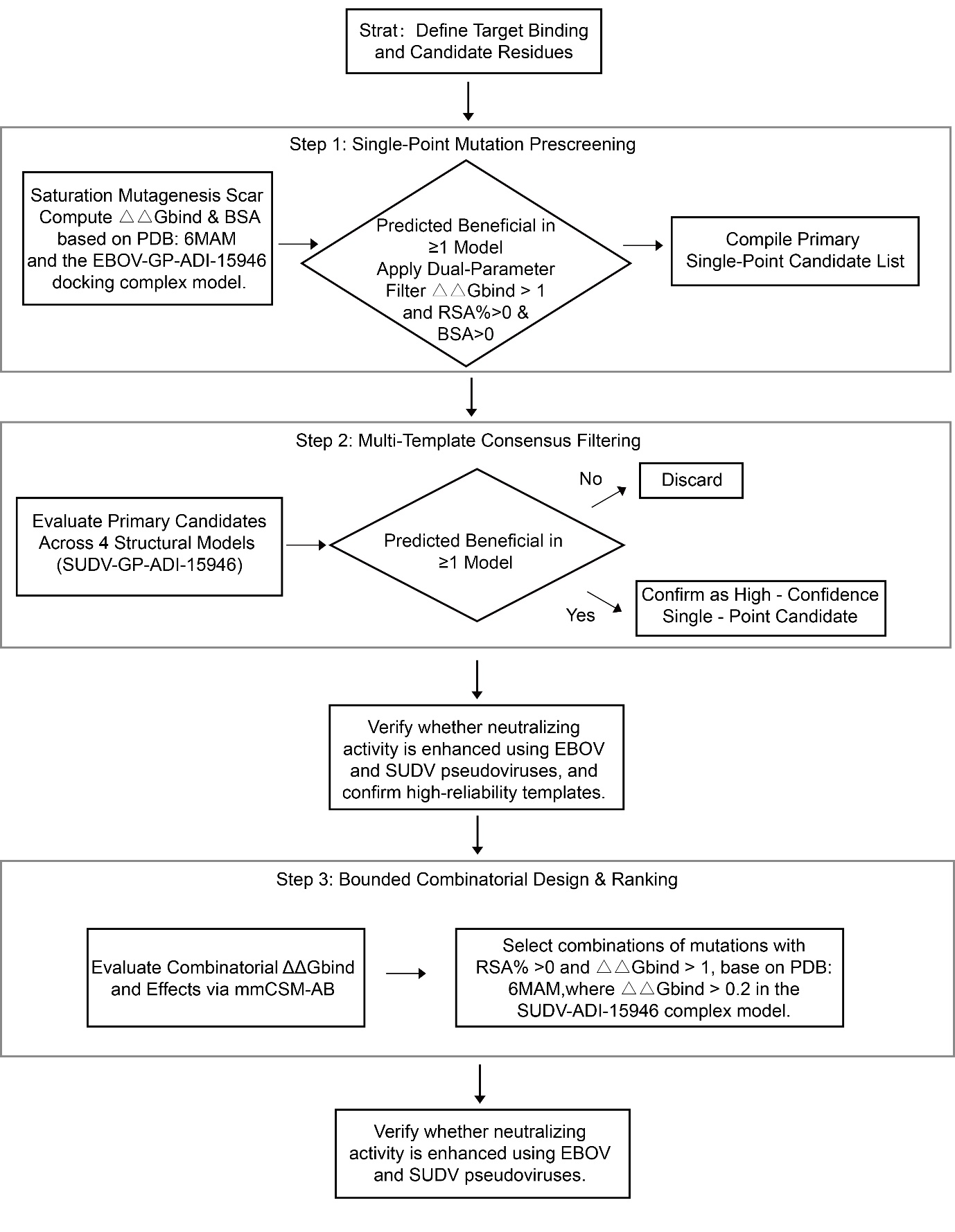
**

**Fig. S12.**

**
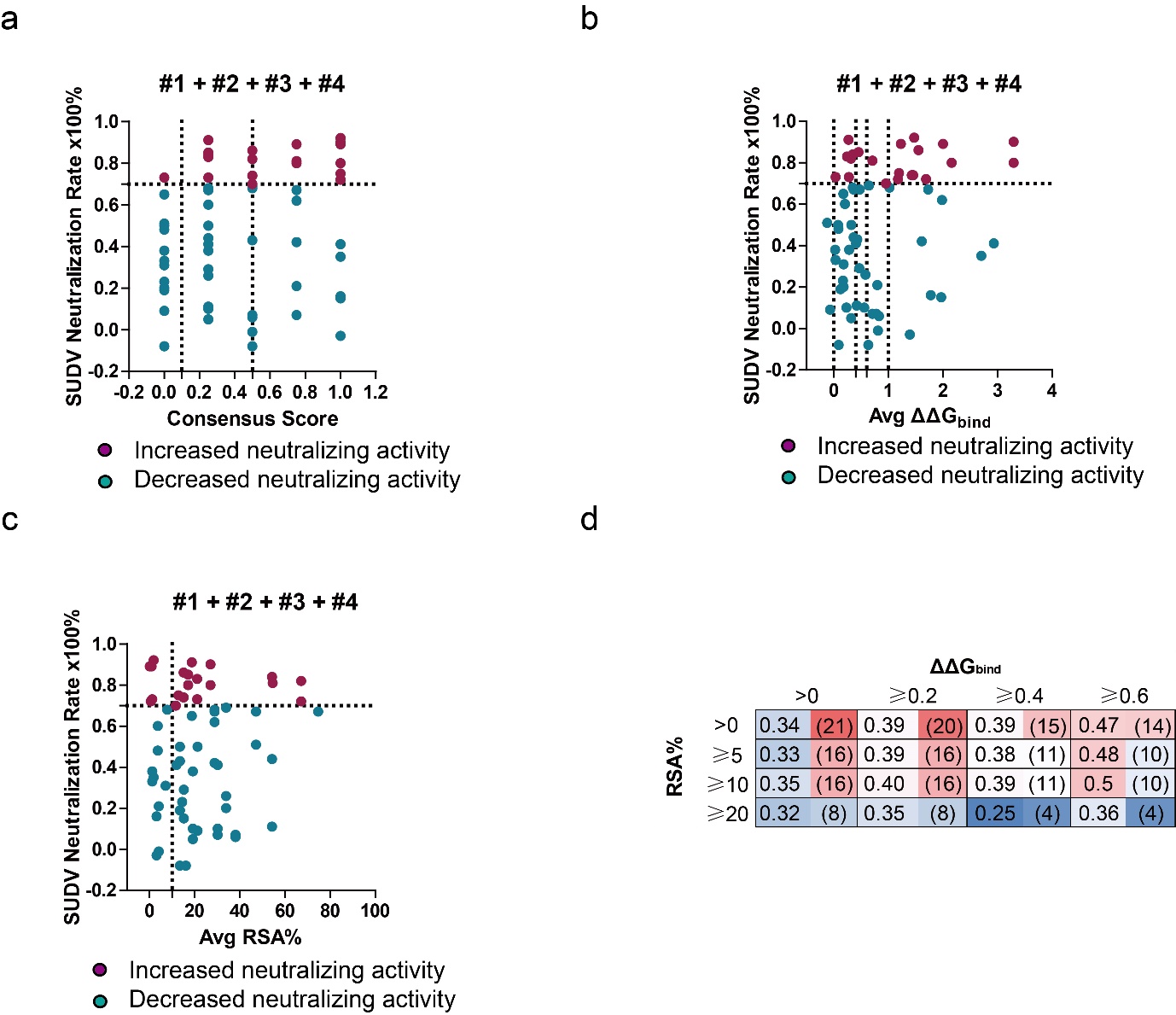
**

***Fig. S12. SUDV-GP-ADI-15946 4 Models Mixed Threshold Sensitivity Analysis.*** *(a) Relationship between multi-template consensus and neutralization. Each predicted ΔΔG_bind_ at this site exceeds 0.6, so we propose averaging the scores and adding 0.25. (b) Relationship between predicted average ΔΔG_bind_ and neutralization. (c) Effect of average RSA% on neutralization. (d) ΔΔG_bind_ and RSA% scan range design, the table contains: Positive rate (Positive count).*

**Fig. S13.**

**
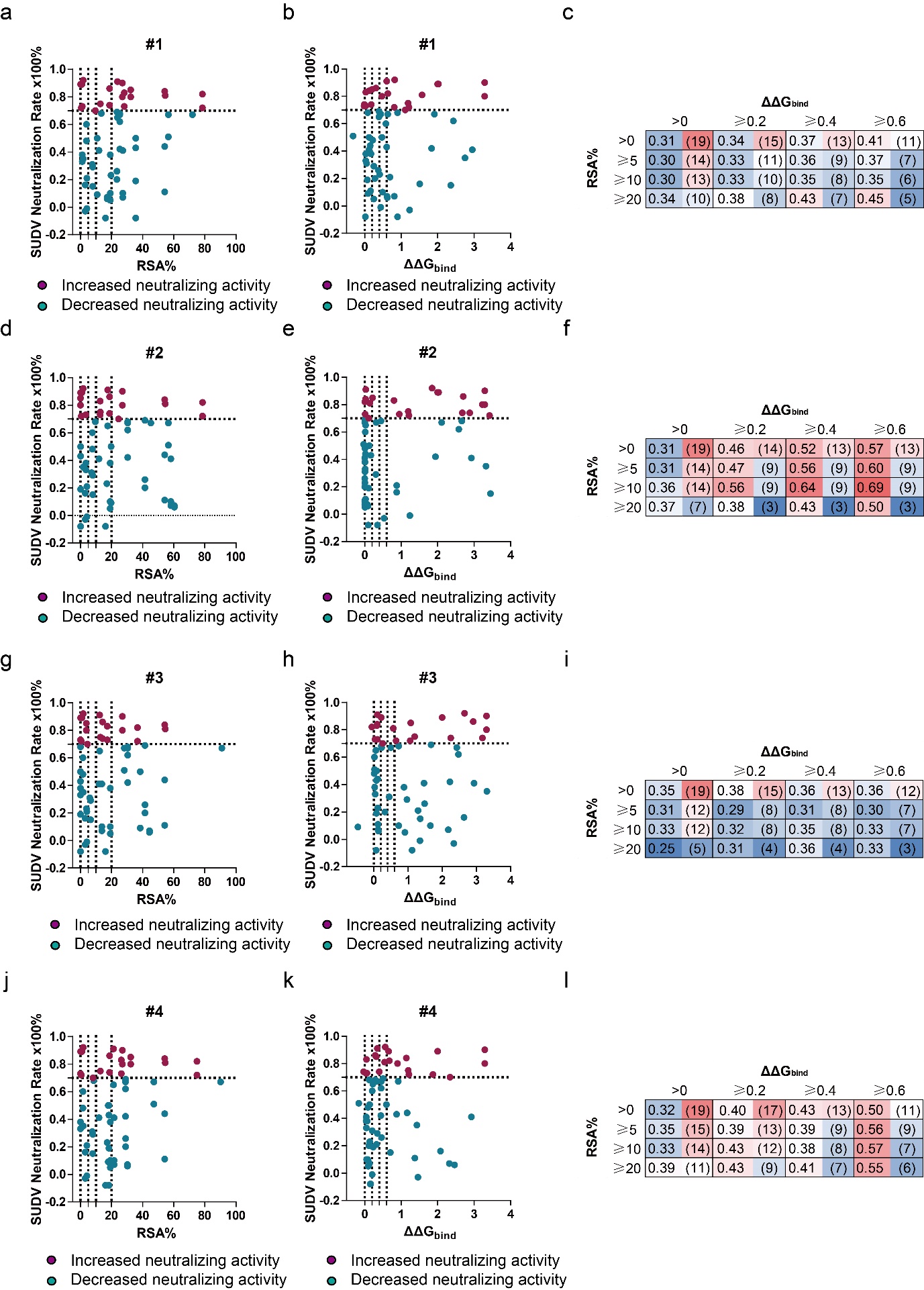
**

***Fig. S13.*** *SUDV-GP-ADI-15946 Single Models Threshold Sensitivity Analysis. (a, d, g, j) Effect of RSA% on neutralization. (b, e, h, k) Relationship between predicted ΔΔG_bind_ and neutralization. (c, f, I, l) ΔΔG_bind_ and RSA% scan range design, the table contains: Positive rate (Positive count).*

**Fig. S14.**

**
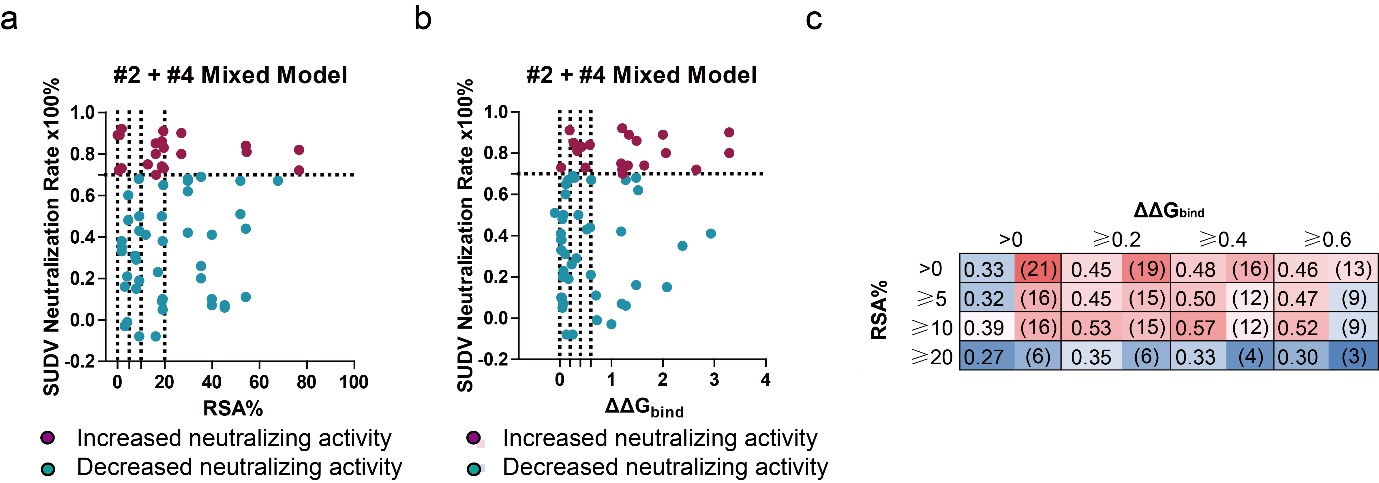
**

***Fig. S14.*** *SUDV-GP-ADI-15946 Mixed Model Threshold Sensitivity Analysis. (a) Effect of average RSA% on neutralization. (b) Relationship between predicted average ΔΔG_bind_ and neutralization. (c) ΔΔG_bind_ and RSA% scan range design, the table contains: Positive rate (Positive count).*

**Fig. S15.**

***Fig. S15****. The π–π stacking interaction formed between antibody light-chain Y52 and GP protein residues P133 and Y543 demonstrates, illustrating the enhanced interfacial stabilization introduced by the S52Y mutation.*

**Fig. S16.**

*
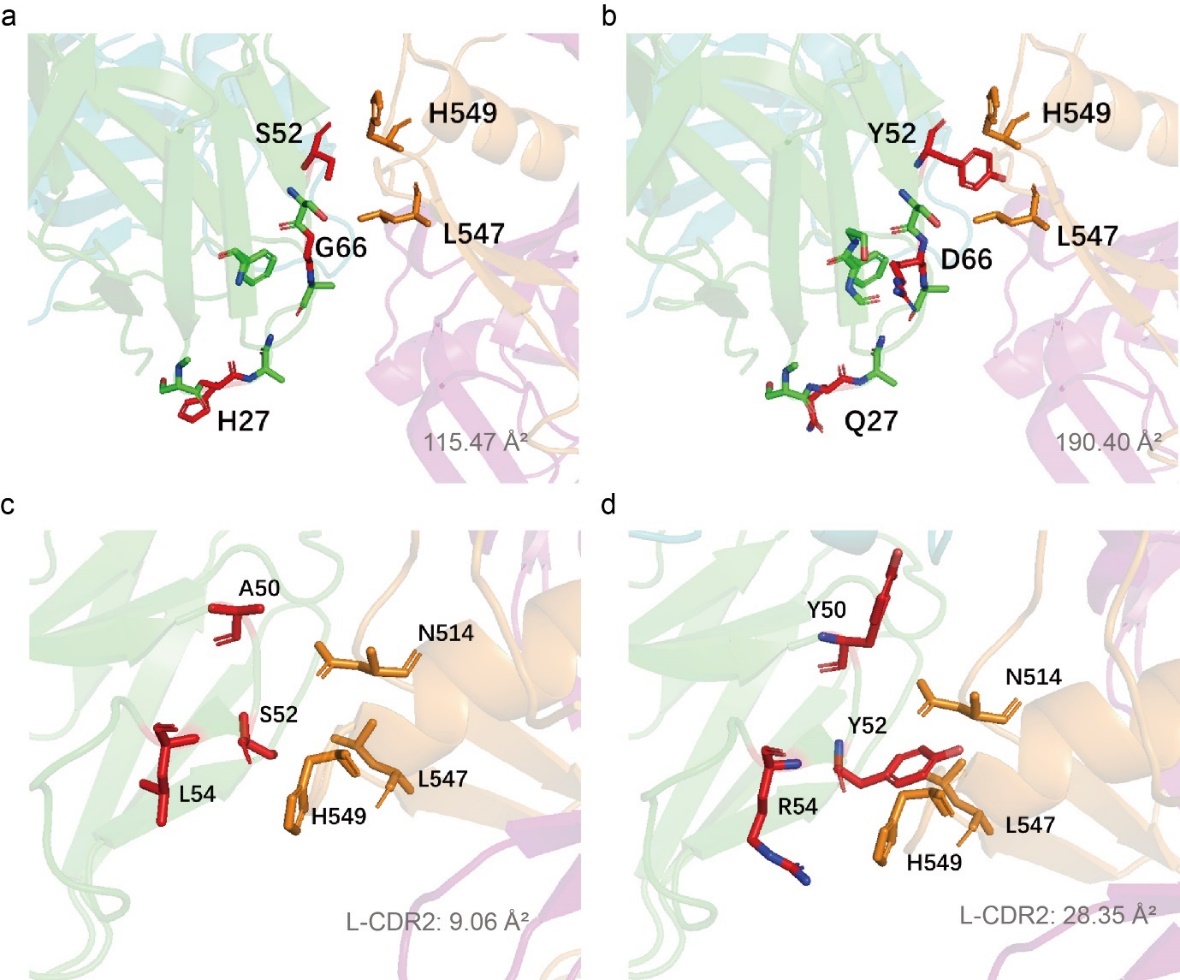
*

***Fig. S16.*** *Analysis of EBOV-GP Protein Interaction Sites with ADI-15946 and Mutant Antibodies. (a) Simulation of key residues H27, S52, and G66 in the EBOV GP-ADI-15946 complex (PDB:6MAM). (b) Simulation of key residues Q27, Y52, and R66 in the EBOV GP-ADI-15946 complex (PDB:6MAM). (c) Simulation of key residues A50, S52, and L54 in the EBOV GP-ADI-15946 complex (PDB:6MAM). (d) Simulation of key residues Y50, Y52, and R54 in the EBOV GP-ADI-15946 complex (PDB:6MAM).*

**Fig. S17.**


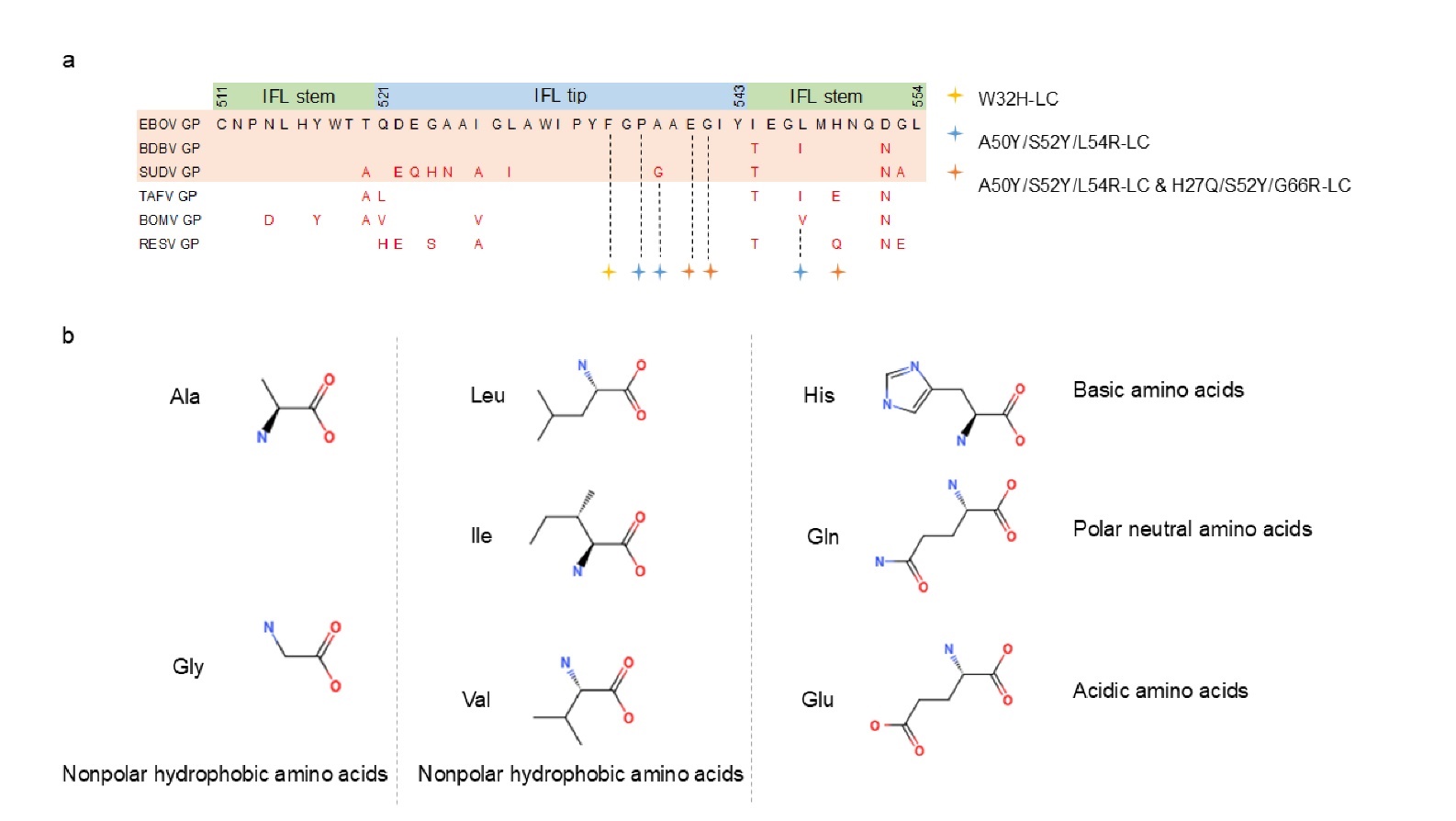


***Fig. S17.*** *Comparison of IFL Amino Acid Sequences Among Six Ebola Viruses. (a) Amino acid sequence alignment of the IFL region across six Ebola viruses. Residues differing from EBOV are highlighted in red, while asterisks indicate antibody binding sites within this region. (b) Comparison of non-conservative amino acid residue types at antibody binding sites.*

**Fig. S18.**

*
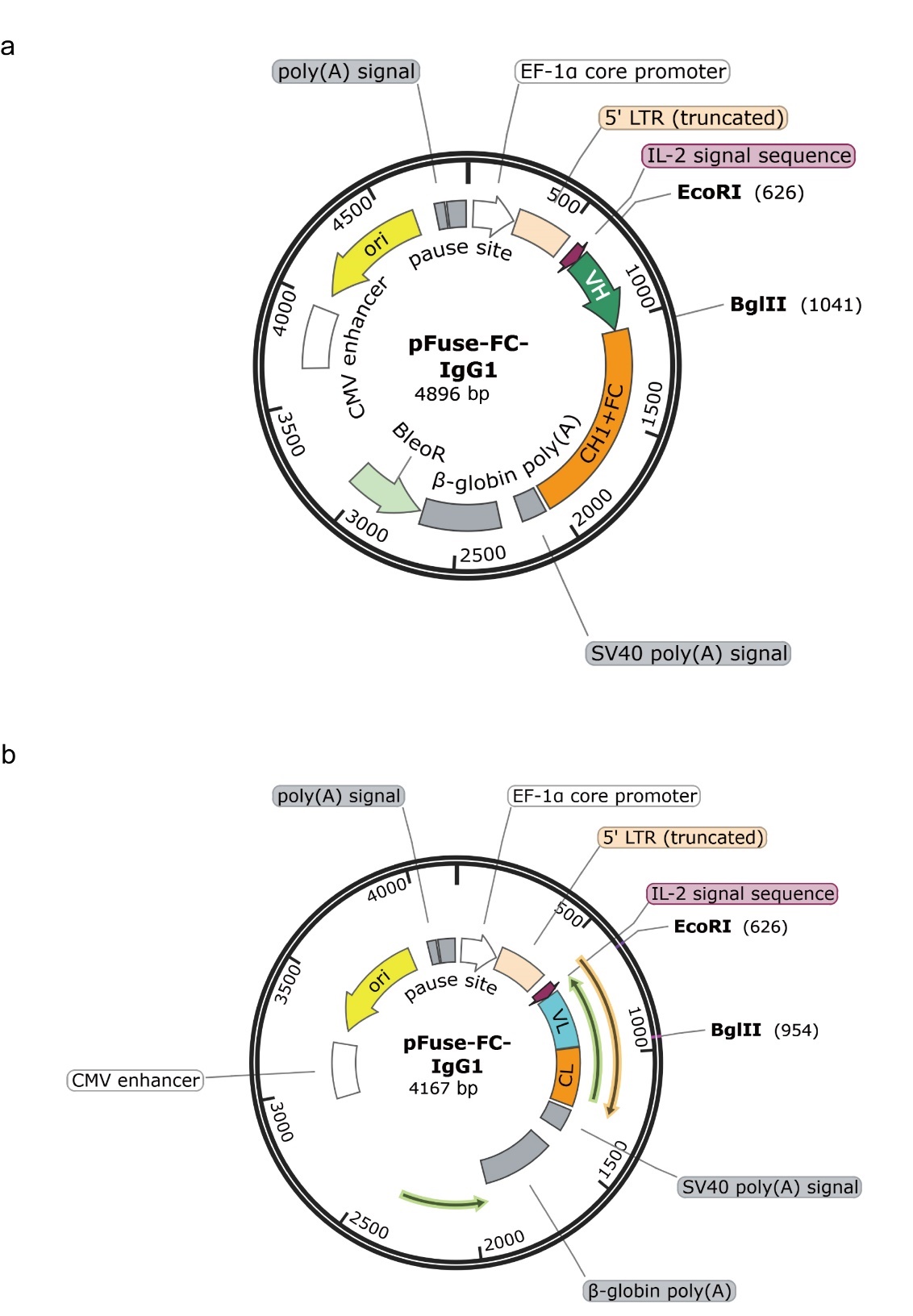
*

***Fig. S18.*** *Schematic of Antibody Expression Constructs. (a) Map of the heavy-chain expression plasmid. (b) Map of the light-chain expression plasmid.*

**Fig. S19.**

*
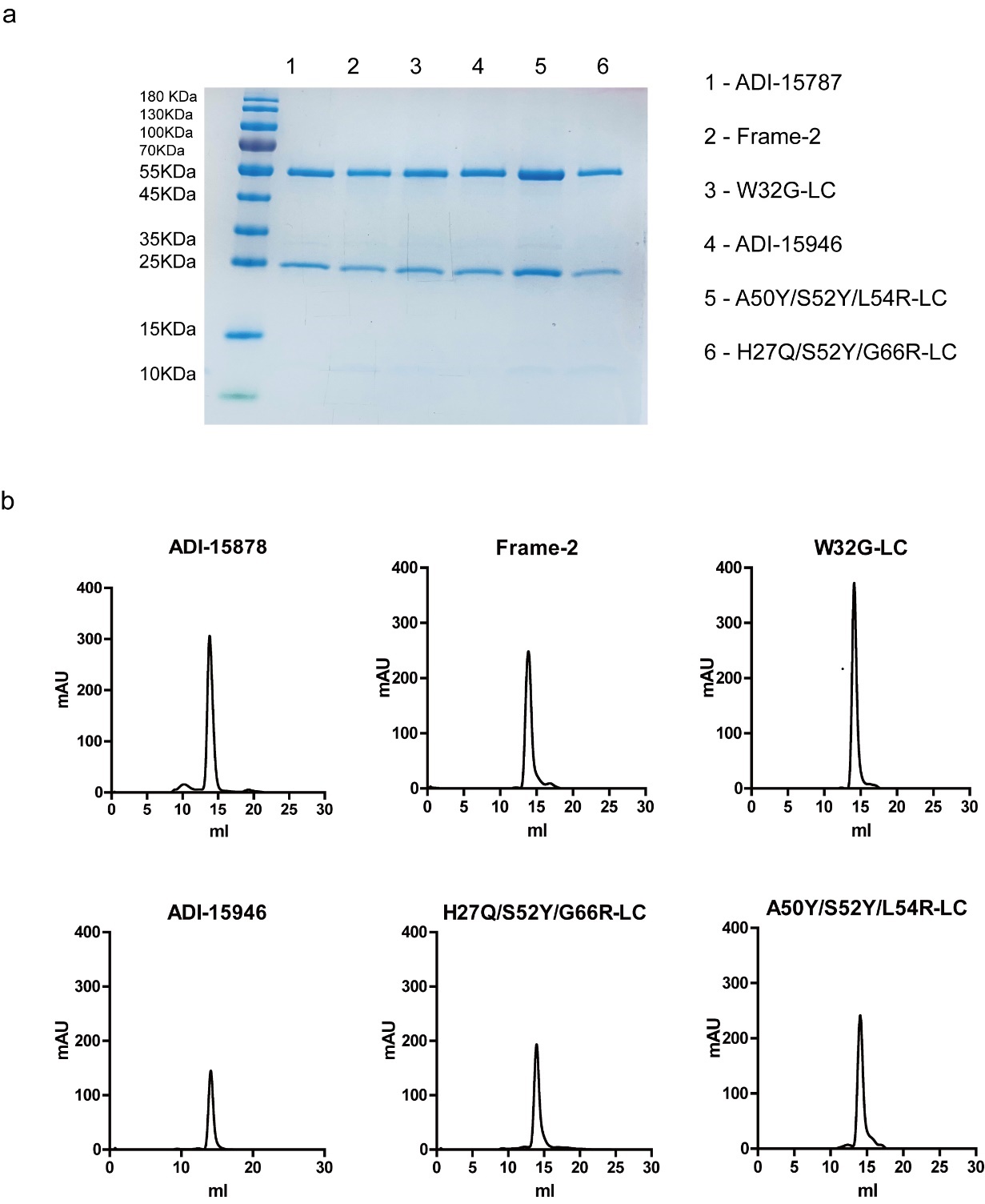
*

***Fig. S19.*** *Analysis of Purified Antibody.* *(a) Sodium dodecyl sulfate-polyacrylamide gel electrophoresis (SDS-PAGE) analysis under reducing conditions. (b) Size-exclusion chromatography (SEC) profile.*

**Table. S1. Computer Prediction of Multiple Point Mutations and Neutralization Assay Results**

| **Mutation Site** | **6MAM** | **#2**  **Model** | **#2 + #4**  **Mixed Model** | **SUDV Neutralization Rate x100%** |
| --- | --- | --- | --- | --- |
| I29Y/A50Y/S52Y-LC | 4.9 | 0.45 | 0.98 | 0.81 |
| A50Y/S52Y/L54R-LC | 4.3 | 1.46 | 0.61 | 0.95 |
| H27Q/S52Y/G66D-LC | 2.9 | 0.37 | 0.27 | 0.96 |
| Y32W/S52Y/L54R-LC | 2.7 | 1.44 | 0.58 | 0.81 |
| S52Y/G64I/G66K-LC | 1.3 | 1.53 | 0.30 | 0.22 |
| S52Y/L54R/G66F-LC | 1.1 | 1.00 | -0.44 | 0.42 |
| S52Y/G66R-LC E103R-HC | 0.3 | -0.41 | -0.69 | 0.75 |
| S30Y/S52Y/G66R-LC | 0.2 | 0.09 | 0.08 | 0.43 |

**Table. S2.** *ADI-15946 and Its Optimized Antibody SPR Assay*

|  | ka (1/Ms) | kd (1/s) | KD (M) | Rmax (RU) |
| --- | --- | --- | --- | --- |
| ADI-15946 | 1.11E+06 | 0.002557 | 2.30E-09 | 16.16 |
| A50Y/S52Y/L54R-LC | 3.69E+06 | 0.001287 | 3.49E-10 | 27.3 |
| H27Q/S52Y/G66R-LC | 1.80E+06 | 4.46E-04 | 2.48E-10 | 28.89 |
